# High-Capacity Be(II) Adsorption by a Multidentate TFP-HEDA Adsorbent: Mechanistic Insight and Statistical Validation

**DOI:** 10.3390/ma19091805

**Published:** 2026-04-28

**Authors:** Gamal M. A. Mahran, Mohamed A. Gado

**Affiliations:** 1 Mining Engineering Department, King Abdulaziz University, Jeddah 21589, Saudi Arabia; 2 Geology Isotopes Department, Research Sector, Nuclear Materials Authority, El-Maadi, Cairo P.O. Box 530, Egypt; mag.nma@yahoo.com

**Keywords:** beryllium recovery, chemisorption mechanism, error analysis, multidentate Schiff base, BeO nanoparticles, statistical model validation

## Abstract

**Highlights:**

**What are the main findings?**

- TFP-HEDA reaches an experimental maximum Be^2+^ adsorption capacity of 163.67 ± 6.42 mg g^−1^, closely matching the Langmuir-predicted value of 163.93 mg g^−1^ (deviation < 0.2%). It ranks among the highest reported adsorbents and is governed by strong inner-sphere chemisorption through Be–O/N coordination (E = 28.87 kJ mol^−1^). It follows Langmuir monolayer behavior (R^2^ = 0.9835) and is optimized to achieve 99% removal efficiency at pH 5 via RSM-CCD (R^2^ = 0.9892).

**What are the implications of the main findings?**

- TFP-HEDA represents a structurally characterized adsorbent demonstrating 99% Be^2+^ removal efficiency under optimized conditions, with nine-cycle regeneration stability from complex aqueous matrices, with potential direct application in nuclear, aerospace, and electronics industrial wastewater treatment, where beryllium poses severe toxicological and environmental risks.
- The multidentate Schiff-base design principle demonstrated here—combining a conjugated TFP aromatic core with flexible N/O donor arms—provides a promising design strategy for porous organic adsorbents incorporating defined hard-donor arrays for selective recovery of high-charge-density metal ions (e.g., Pb^2+^, Cd^2+^, Cr^6+^) with tunable selectivity.
- The nine-cycle adsorption–desorption stability of TFP-HEDA, confirmed by post-cycling XPS and FTIR characterization demonstrating full retention of the imine C=N, phenolic C–OH, and carbonyl C=O coordination framework (Section 3.5.1), establishes the material’s practical regenerability and identifies it as a candidate for column-based continuous-flow Be^2+^ recovery from nuclear processing and aerospace manufacturing effluents.
- The comprehensive RSM-CCD statistical validation framework, encompassing 20 independent diagnostic tests with a 100% pass rate, demonstrates a 20-parameter RSM-CCD validation approach applicable to adsorption process optimization, demonstrating that initial Be(II) concentration and adsorbent dosage jointly govern 66.76% of process variance, enabling reliable industrial scale-up design without additional experimentation.
- The demonstrated conversion of Be(II)-loaded TFP-HEDA into high-purity BeO nanostructures following desorption establishes a circular, value-added recovery pathway that transforms hazardous beryllium waste into a functional advanced ceramic material, providing a recovery pathway consistent with principles of circular resource management.

**Abstract:**

The selective removal of beryllium from aqueous matrices remains a critical environmental and industrial challenge due to beryllium’s extreme toxicity, strong hydration chemistry, and the difficulty of separating Be^2+^ from chemically similar cations such as Al^3+^. In this study, a novel multidentate Schiff-base porous organic adsorbent, TFP-HEDA, was synthesized by condensation of 2,4,6-trihydroxybenzene-1,3,5-tricarbaldehyde (TFP) with N-(2-hydroxyethyl)ethylenediamine (HEDA) followed by urethane post-functionalization and systematically characterized by FTIR, ^1^H/^13^C NMR, MALDI-TOF MS, elemental analysis, BET surface area analysis (617 m^2^ g^−1^), PXRD, and XPS. Batch adsorption experiments demonstrated rapid Be^2+^ uptake, achieving 90% removal within 20 min and equilibrium within 30 min. Among the isotherm models evaluated, the Langmuir model yielded the highest statistical consistency (R^2^ = 0.9835, RMSE = 5.15 mg g^−1^, χ^2^ = 1.137) with a predicted maximum adsorption capacity of 163.93 mg g^−1^ agreeing closely with the experimental value of 163.67 ± 6.42 mg g^−1^ (deviation < 0.2%); this mathematical adequacy is interpreted as compatibility with a finite, saturable set of inner-sphere coordination sites rather than confirmation of a flat, energetically uniform surface, with chemisorption independently and more rigorously established by Dubinin–Radushkevich analysis (E = 28.87 kJ mol^−1^) and post-adsorption FTIR and XPS evidence. Dubinin–Radushkevich analysis confirmed a chemisorption mechanism with mean adsorption energy E = 28.87 kJ mol^−1^, consistent with inner-sphere Be^2+^–O/N coordination. Process optimization using response surface methodology based on a central composite design achieved 99% Be^2+^ removal at pH 5, an adsorbent dose of 60 mg/20 mL, and a contact time of 30 min (R^2^ = 0.9892). Post-adsorption FTIR, XPS, BET, and TGA characterization confirmed framework integrity and the inner-sphere multidentate coordination mechanism. TFP-HEDA retained 82.4% of its initial capacity after nine adsorption–desorption cycles, demonstrating practical regenerability for Be^2+^ recovery applications.

## 1. Introduction

Beryllium is a strategically critical metal extensively employed in aerospace structural components, nuclear reactor moderators and reflectors, X-ray window fabrication, and high-performance electronic devices [1,2]. Global beryllium production reached approximately 500 tonnes annually by 2018, with primary sources from bertrandite (Be_4_Si_2_O_7_(OH)_2_) processing in the United States and beryl (Al_2_Be_3_Si_6_O_18_) mining in South America. Despite its technological value, beryllium and its compounds are classified as Group 1 carcinogens (IARC). They are regulated at workplace air concentration limits of 0.05 μg m^−3^ (OSHA 2020), making the management of beryllium-bearing process streams a matter of both environmental and occupational health urgency [3,4].

Beryllium and its compounds are widely recognized for their exceptional combination of properties, which makes them indispensable in numerous high-technology applications. Beryllium ceramics are particularly valued as efficient heat sinks due to their excellent thermal conductivity. Beryllium-containing alloys are utilized to enhance the performance of various engineered products, including automotive sensors, fatigue-resistant springs, undersea communication cables, and specialized non-sparking tools designed for use in hazardous or explosive environments. In engineering and nuclear science, beryllium plays a unique role as an effective neutron moderator, reflector, and shielding material due to its outstanding neutron-scattering capability. It is also employed as an inner lining for fusion reactor chambers and as an additive in fission fuels to improve coolant efficiency.

However, alongside these technological advantages, beryllium presents significant health and handling challenges. It is classified as a toxic and potentially carcinogenic element. Inhalation of fine beryllium dust or fumes can sensitize between 1% and 15% of individuals exposed to it. This may result in the development of chronic beryllium disease (CBD), a severe, immune-driven lung disorder marked by granulomatous inflammation and worsening respiratory impairment. These health hazards, combined with the element’s inherent brittleness and relatively high production costs, underscore the importance of implementing stringent safety protocols.

Despite these risks, the continued high demand for beryllium in advanced technologies has spurred intensive research efforts to develop safer processing and handling methods, improve protective occupational standards, and explore innovative approaches in beryllium metallurgy and chemistry [5,6,7].

Beryllium is usually obtained by leaching beryl ore and precipitating beryllium hydroxide, a crucial intermediate in its production that influences the quality of subsequent products. Beryl ore is highly resistant to most mineral acids under normal conditions, except hydrofluoric acid. Thus, the extraction typically begins with fusion using KOH and KCO_3_ to degrade the beryl crystal structure, tracked by rapid cooling in water [8]. The material is then reheated, ground, and dissolved in sulfuric acid [9]. In the sulfate method, silica is filtered out, aluminum is precipitated with alum, and the leach liquor is subjected to solvent extraction to produce a beryllium-rich solution [10]. As part of the fluoride-based extraction process, beryl is subjected to a high-temperature sintering step, where it reacts with fluorinating reagents such as ammonium fluoride (NH_4_F) and ammonium bifluoride (NH_4_HF_2_) to enable the breakdown and subsequent chemical transformation of the mineral matrix. These chemicals specifically convert the beryllium in the ore into sodium beryllium fluoride solution [11,12,13].

The sulfuric acid solution primarily contains aluminum, beryllium, and various impurities. Ammonia liquor is generally employed to precipitate beryllium as hydroxide, whereas most ammonium alum stays dissolved. Since beryllium and aluminum are chemically very similar—both amphoteric and forming complexes with comparable formation constants [14,15] aluminum remains a significant impurity. It is not easy to eliminate through simple precipitation or crystallization methods [16,17].

Various methods have been investigated to purify beryllium hydroxide. One technique involves washing the precipitate with an EDTA solution to form soluble aluminum complexes, yielding pure beryllium hydroxide [16]. Another method converts beryllium hydroxide into basic beryllium acetate using acetic acid, which can then be selectively extracted with chloroform. Additionally, solvent extraction has been extensively examined for separating beryllium from aluminum, such as the use of di-(2-ethylhexyl) phosphoric acid (HDEHP) to extract Be(II) and Al(III) from sulfate solutions; this technique is slow and highly pH-dependent [18,19,20,21].

Other extraction methods employ acidic extractants, such as Cyanex 272, which has been used to leach solutions from saturated beryl ore, followed by separation of beryllium and aluminum via sulfuric acid stripping. Neutral extractants, such as Cyanex 921, form complexes like [Be(OH)_2_·2(Cyanex 921)] and [AlCl_3_·3(Cyanex 921)], which are used for Be–Al separation in both cyclohexane and extraction chromatography within hydrochloric acid solutions. Alkaline systems using Adogen 464 depend on anion exchange, where beryllium hydroxide dissolves in ammonium bicarbonate–carbonate to form (R_4_N)_2_Be(CO_3_)_2_ for extraction. However, challenges arise in recycling ammonia and CO_2_, as well as in co-extracting boron [22,23,24,25].

Because of the chemical similarities between beryllium and aluminum, most extractants tend to co-extract both elements. Naphthenic acid, commonly used in rare-earth chloride systems, has proven effective for separating aluminum and rare-earth metals at high Fe and Al levels [26]. Although its use in beryllium systems is still limited domestically, it is effective in these applications.

Porous covalently linked organic architectures have become an important family of tailor-made sorbents for liquid phase separations. Built from light elements such as C, H, O, N, and B and stitched together by strong covalent linkages, these materials can offer high internal surface area, tunable pore environments, and low framework density, which is difficult to achieve with conventional inorganic adsorbents or amorphous polymeric resins. In many systems, extended π conjugated backbones bearing heteroatom donor sites create well-defined binding pockets where metal ions are engaged through a combination of coordination, hydrogen bonding, electrostatic forces, and secondary π interactions [27]. Because the organic building blocks can be selected and arranged with molecular precision, both the geometry of the cavities and the electronic character of the binding sites can be tuned, which is crucial for discriminating between competing cations in complex aqueous matrices [28,29,30].

Within this broader design landscape, the TFP-HEDA material was conceived as a multifunctional porous organic sorbent. TFP-HEDA arises from the condensation of a triformylphloroglucinol (TFP) core with ethylenediamine-type segments derived from hydroxyethyl diamines, generating an extended aromatic scaffold decorated with multiple hydroxyl, imine/amine, and amide functionalities. The TFP unit contributes a conjugated phenolic/β dicarbonyl motif that can deprotonate and donate electron density, while the HEDA fragments introduce flexible aliphatic linkers terminating in nitrogen and carbonyl donors. Together, these features create a dense array of hard donor centers (O and N) distributed around accessible pores, providing a highly chelating environment for small, highly charged metal ions such as Be(II) [31]. The residual ester and vinyl groups at the periphery further provide handles for post-synthetic modification or immobilization, enabling future integration of TFP-HEDA into composite or supported systems, if required [32,33].

Previous studies on porous organic adsorbents have shown that directly incorporating polar and chelating groups such as –OH, –COOH, –SO_3_H, and –NH_2_ into the framework backbone greatly enhances metal ion uptake, improves selectivity, and facilitates regeneration, particularly in applications related to metal recovery from aqueous waste, environmental cleanup, and preconcentration before instrumental analysis [34,35,36]. TFP-HEDA follows this design principle: phenolic hydroxyls and carbonyl oxygens provide primary coordination sites, while nearby amine and imine nitrogens act as secondary donors, enabling multidentate binding of Be(II) within confined microenvironments. In aqueous solution, partial deprotonation of phenolic sites and amide/imine tautomerization are expected to promote inner sphere complex formation, whereas the remaining –OH and –NH groups sustain hydrogen bond networks that assist in pre-organizing hydrated Be(II) species near the surface. This cooperative mechanism—combining hard donor coordination, hydrogen bonding, and electrostatic attraction—underpins the anticipated high affinity of TFP-HEDA toward Be(II) even in the presence of other multivalent cations [37,38].

Despite these developments, a critical gap persists in the literature: the vast majority of reported Be^2+^ adsorbents—including calcium sulfate hydrate-encapsulated silica (147.55 mg g^−1^), MXene/nZVI/FH composites (95.2 mg g^−1^), modified sulfonated chitosan (40 mg g^−1^), and modified polystyrene (16 mg g^−1^)—were designed without a molecular-level rationale linking donor-atom hardness, coordination geometry, and pore architecture specifically to the unique coordination requirements of Be^2+^. None of these materials simultaneously address the three requirements that define a practically viable Be^2+^ adsorbent: geometric selectivity for tetrahedral coordination, inner-sphere chemisorption confirmed by post-adsorption characterization, and statistically validated multi-variable process optimization. The present work addresses this gap by reporting TFP-HEDA—a porous Schiff-base adsorbent whose phenolate-O, imine-N, and carbonyl-O donor array was specifically designed through HSAB principles to match the hard Lewis acid character and exclusive tetrahedral coordination preference of Be^2+^—validated by comprehensive post-adsorption FTIR, XPS, BET, and TGA characterization alongside RSM-CCD process optimization.

## 2. Materials and Methods

### 2.1. Chemicals and Solvents

2,4,6-trihydroxybenzene-1,3,5-tricarbaldehyde (TFP) (≥99%) and N-(2-hydroxyethyl)ethylenediamine mesitylene and 1,4-dioxane were procured from Sigma-Aldrich (Merck KGaA, Darmstadt, Germany)). Aqueous acetic acid and Methacryloyloxyethyl isocyanate were procured from Fisher Scientific (Pittsburgh, PA, USA). Beryllium sulfate, BeSO_4_·4H_2_O, was obtained from Fluka (Sigma-Aldrich Chemie GmbH, Buchs, Switzerland). Chrome Azurol S and other spectrophotometric reagents were obtained from Sigma-Aldrich (Merck KGaA, Darmstadt, Germany). All chemicals were used without further purification.

### 2.2. Instruments and Apparatus

In this study, a series of advanced analytical techniques was used to thoroughly characterize the physicochemical properties and adsorption performance of the synthesized TFP-HEDA adsorbent for removing beryllium ions from water solutions. The specific surface areas of TFP-HEDA and HEDA were determined using the Brunauer–Emmett–Teller (BET) method from N_2_ adsorption–desorption isotherms measured at 77 K on a Micromeritics ASAP 2460 instrument (Micromeritics Instrument Corp., Norcross, GA, USA). Samples (approximately 100 mg) were degassed at 100 °C under dynamic vacuum (<10 μmHg) for 12 h before measurement. The BET surface area was calculated from adsorption data in the relative pressure range 0.05 ≤ P/P_0_ ≤ 0.30. The pore size distribution was independently determined by Non-Local Density Functional Theory (NLDFT) applied to the full N_2_ adsorption branch, and the total pore volume was estimated from the N_2_ uptake at P/P_0_ = 0.99. The Barrett additionally determined the mesopore size distribution using the Joyner–Halenda (BJH) method applied to the desorption branch of the N_2_ isotherm.

For pH monitoring and adjustment during the experiments, a Digimed DM21 digital pH meter (Digimed, São Paulo, Brazil) with an accuracy of ±0.1 was used. Batch adsorption experiments were conducted by combining weighed amounts of TFP-HEDA adsorbent (20–200 mg) with 20 mL volumes of Be^2+^ solution in 50 mL polypropylene vessels, sealed and agitated on a Vibromatic 384 orbital shaker (Vibromatic Equipamentos Científicos Ltd.a., São Paulo, Brazil) at 200 rpm under controlled temperature conditions (25–45 °C). Initial Be^2+^ concentrations ranged from 10 to 500 mg L^−1^; solution pH was adjusted in the range 1–9 using 0.1 mol L^−1^ HCl or NaOH; and contact times were varied from 2 to 60 min. After equilibration, suspensions were filtered through 0.45 μm PTFE membrane filters, and the filtrate was analyzed for residual Be^2+^ concentration by the Chrome Azurol S spectrophotometric method at 570 nm. After reaching equilibrium, the remaining concentrations of Na^+^, Li^+^, K^+^, Ca^2+^, and Mg^2+^ ions in the supernatant were measured using inductively coupled plasma optical emission spectroscopy (ICP-OES, PerkinElmer Avio 220) (PerkinElmer Inc., Waltham, MA, USA).

Structural and functional group analysis of the materials was performed using Fourier transform infrared spectroscopy (FTIR) on a GascoJapan FTIR 4100 spectrometer (Gasco Japan, Tokyo, Japan), employing potassium bromide (KBr) pellets for sample preparation. ^1^H and ^13^C NMR spectra were recorded on a Bruker Mercury 400 spectrometer (^1^H resonance frequency: 400 MHz; ^13^C resonance frequency: 100.6 MHz) (Bruker BioSpin GmbH, Rheinstetten, Germany) at 293 K using DMSO-d_6_ as solvent, with tetramethylsilane (TMS) as internal reference, with the samples dissolved in DMSO-d_6_. Chemical shifts (δ) are reported in parts per million (ppm), and coupling constants (J) are given in hertz (Hz). Matrix-assisted laser desorption/ionization time-of-flight mass spectrometry (MALDI-TOF MS) (Bruker Daltonics GmbH & Co. KG, Bremen, Germany) was performed on a Bruker Autoflex Speed mass spectrometer (Bruker Daltonics, Bremen, Germany) equipped with a nitrogen laser (λ = 337 nm). Sample preparation followed the standard dried-droplet co-crystallization procedure: TFP-HEDA was dissolved in DMSO at a concentration of 1 mg mL^−1^ and mixed in a 1:10 (*v*/*v*) ratio with a saturated solution of α-cyano-4-hydroxycinnamic acid (CHCA) as an organic matrix, prepared in acetonitrile/water/trifluoroacetic acid (50:49.9:0.1, *v*/*v*/*v*). One microliter of the analyte–matrix mixture was deposited onto a stainless-steel MALDI target plate and allowed to air-dry at room temperature before analysis. Spectra were acquired in positive-ion reflectron mode under high-vacuum conditions (<1 × 10^−6^ mbar), with an accelerating potential of ±20 kV and a 40 ns delayed extraction to improve mass resolution. Each reported spectrum represents the accumulated signal from 30 laser shots, with laser fluence adjusted just above the ionization threshold to minimize in-source fragmentation. Two-point external calibration using a standard mixture spanning the 200–2000 Da mass range was performed prior to sample acquisition.

X-ray photoelectron spectroscopy (XPS) measurements were performed on a [Thermo Fisher Scientific KAlpha instrument] spectrometer (Thermo Fisher Scientific, Waltham, MA, USA) using monochromatic Al Kα radiation (1486.6 eV) under ultra-high vacuum conditions (<10^−9^ mbar). Survey spectra were acquired over the 0–1300 eV range, and high-resolution spectra were recorded for C 1s, N 1s, O 1s, and Be 1s regions. All binding energies were referenced to the adventitious C 1s peak at 284.8 eV to correct for surface charging. Peak fitting was performed using Gaussian–Lorentzian profiles with a Shirley-type background subtraction.

Thermogravimetric analysis (TGA) was performed using a TA Instruments Q500 thermogravimetric analyzer (TA Instruments, New Castle, DE, USA). Samples of 5–10 mg were placed in alumina crucibles and heated under a flowing nitrogen atmosphere (60 mL/min purge, 40 mL/min balance) from 25 °C to 860 °C at a heating rate of 10 °C/min. TGA measurements were conducted on the HEDA precursor, as-synthesized TFP-HEDA, and Be^2+^-loaded TFP-HEDA-Be(II) following equilibrium adsorption and vacuum-drying at 50 °C for 12 h.

X-ray diffraction (XRD) analysis was conducted using a Philips PW-3710 unit and PW-1830 generator (Philips Analytical, Almelo, The Netherlands), with Cu target radiation at 40 kV and 30 mA and a Ni filter. Surface morphologies were examined through environmental scanning electron microscopy (ESEM, Model EXL 30, (FEI Company, Hillsboro, OR, USA)). Beryllium concentrations were determined spectrophotometrically using the Chrome Azurol S method, with the absorption peak at 570 nm measured on a LABOMED double-beam spectrophotometer (USA) (Labomed Inc., Los Angeles, CA, USA) [39].

### 2.3. Adsorption Experiment of TFP-HEDA

In a representative adsorption experiment, 60 mg of TFP-HEDA was added to 20 mL of Be^2+^ solution (initial concentration 250 mg L^−1^, pH 5.0) in a 50 mL polypropylene vessel. The suspension was agitated at 200 rpm and 25 °C for 30 min, then filtered through a 0.45 μm PTFE membrane. For systematic parameter studies, the following variables were examined individually while holding all other parameters at their reference values (dose 60 mg/20 mL; Be^2+^ = 250 mg L^−1^; pH 5; t = 30 min; T = 25 °C): adsorbent dose (20–200 mg/20 mL), initial Be^2+^ concentration (10–500 mg L^−1^), solution pH (1–9), contact time (2–60 min), and temperature (25–45 °C). The mixture was shaken in the thermostatic water bath, and then the solution was separated by a membrane filter (0.45 μm). ICP-OES was employed to measure metal ion levels in the filtered solution. The evaluation of adsorption performance is in Equations (1)–(3).

(1) $$Q=(C_{o}-C_{e})\times \frac{V}{m}$$

(2) $$E=\left(\frac{C_{o}-C_{e}}{C_{o}}\times 100\%\right)$$

(3) $$K_{d}=\frac{C_{o}-C_{e}}{C_{o}}\times \frac{V}{m}$$

Q_e_ (mg g^−1^) denotes the equilibrium sorption amount—the mass of Be^2+^ adsorbed per unit mass of TFP-HEDA at equilibrium—and should not be confused with adsorption capacity, which refers exclusively to the maximum sorption amount at saturation (q_max_, mg g^−1^) derived from isotherm models such as the Langmuir equation. E (%) denotes the removal efficiency, as defined in Equations (1) and (2). Distribution coefficient (K_d_), initial concentration (C_o_, mg L^−1^), and equilibrium concentration (C_e_, mg L^−1^) refer to the initial and equilibrium levels of Be(II). V (mL) indicates the volume of the aqueous solution phase, while *m* (g) denotes the mass of the dry adsorbent.

In pH-dependent experiments of TFP-HEDA with Be(II), solutions of Be(II) in the pH range of 1–8 were prepared. The pH values of Be(II) solutions with an initial concentration of 250 mg L^−1^ were adjusted using HCl or NaOH solutions.

Before the RSM-CCD experimental design, systematic one-factor-at-a-time (OFAT) screening experiments were conducted across broad ranges of variables to identify approximate optimal points and define boundaries for the multivariate design space. pH was tested from 1 to 9, adsorbent dose from 20 to 200 mg per 20 mL, and contact time from 2 to 60 min. These screening results indicated pH 5, 60 mg of adsorbent, and 30 min of contact time as the approximate ideal conditions, which were then used as the central point values for the RSM-CCD, with ±α-star points defining the coded variable boundaries.

All parametric batch adsorption experiments were conducted using synthetic BeSO_4_·4H_2_O solutions to enable rigorous mechanistic characterization; the practical applicability of TFP-HEDA to complex industrial matrices is demonstrated through both a comprehensive tolerance limit evaluation (24 coexisting species, Section 3.5) and direct application to real beryllium-containing sludge leachate (Section 3.8).

### 2.4. Desorption Experiments

For desorption studies, TFP-HEDA was first loaded with Be^2+^ by equilibration with a 250 mg L^−1^ Be^2+^ solution (pH 5.0, 60 mg adsorbent/20 mL, 200 rpm, 30 min, 25 °C). The Be^2+^-loaded adsorbent was separated by filtration through a 0.45 μm PTFE membrane, rinsed three times with deionized water (3 × 5 mL) to remove surface-adsorbed but uncoordinated ions, and air-dried at 50 °C under vacuum for 2 h. The following eluents were systematically screened to identify the most effective desorption agent: deionized water, 0.1 mol L^−1^ HCl, 0.1 mol L^−1^ HNO_3_, 0.1 mol L^−1^ H_2_SO_4_, 0.1 mol L^−1^ EDTA (pH 5), neutral urea (0.4 mol L^−1^), and acidified urea (0.4 mol L^−1^ urea + 0.25 mol L^−1^ H_2_SO_4_). Desorption was performed by contacting 60 mg of Be^2+^-loaded TFP-HEDA with 20 mL of each eluent in a sealed 50 mL polypropylene vessel at 25 °C and 200 rpm for 5–60 min. The optimal eluent (0.4 mol L^−1^ urea + 0.25 mol L^−1^ H_2_SO_4_) was identified as described in Section 3.5 and used for all subsequent regeneration cycles. After each desorption step, the adsorbent was washed with deionized water (3 × 5 mL), pH-readjusted to 5.0, and reused in the next adsorption cycle. Nine consecutive adsorption–desorption cycles were completed to assess regeneration stability. Desorption efficiency (DE%) was calculated according to Equation (4), where C_d_ (mg L^−1^) is the Be^2+^ concentration in the desorption solution, V_d (mL) is the desorption volume, m (g) is the adsorbent mass, and q (mg g^−1^) is the equilibrium sorption amount from the preceding adsorption step. The desorption efficiency was considered using Equation (4).

(4) $$DE(\%)=\frac{C\times V}{q\times m}$$

where C is the concentration of metal ions in the solution (mg L^−1^), V is the volume of the desorbed solution (mL), m is the mass of the TFP-HEDA adsorbent (g), and q denotes the amount of metal ions adsorbed per unit mass of resin (mg g^−1^).

### 2.5. Reaction Description (Detailed Dissection)

**Scheme 1:**
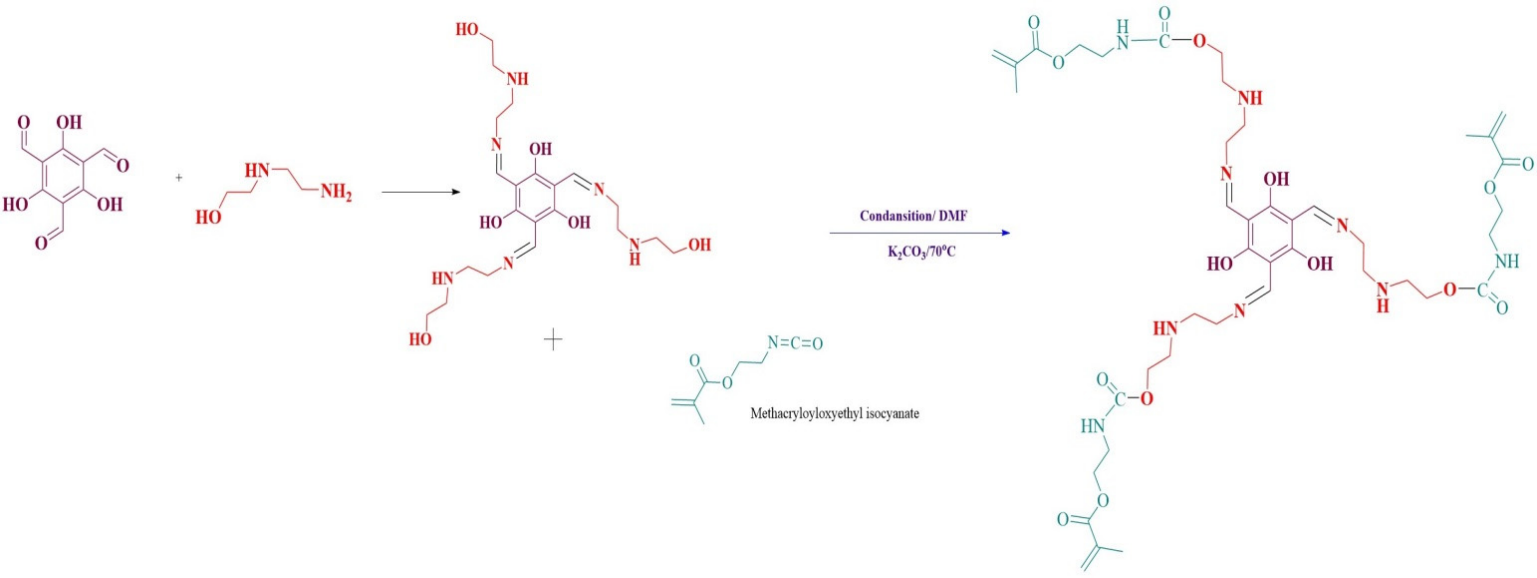
Preparation of TFP-HEDA adsorbent.

The preparation of the methacrylate-functionalized Schiff base ligand TFP-HEDA Scheme 1 follows a two-step pathway, enabling structural stability and functionalization. Initially, 2,4,6-trihydroxybenzene-1,3,5-tricarbaldehyde (TFP) undergoes a condensation reaction with N-(2-hydroxyethyl)ethylenediamine in a mixed solvent system of mesitylene and 1,4-dioxane, catalyzed by aqueous acetic acid, where the amine groups nucleophilically attack the aldehyde carbonyls to form robust imine (C=N) linkages, yielding a red Schiff base intermediate adorned with hydroxyethyl groups. The reaction mixture is sonicated to achieve homogeneity, degassed to remove dissolved gases, and heated to 120 °C for 72 h to drive the condensation to completion. After cooling, the precipitated product is isolated by filtration, washed thoroughly with ethanol, acetone, and dichloromethane to remove unreacted residues, then Soxhlet-extracted with ethanol for 24 h, and dried under vacuum at 70 °C, affording the intermediate as a fine red powder. In the subsequent post-functionalization step, 100 mg of the dried Schiff base is dispersed in anhydrous DMF under a nitrogen atmosphere, where potassium carbonate acts as a mild base to activate the terminal hydroxyl groups for nucleophilic attack. Methacryloyloxyethyl isocyanate is then added dropwise, allowing the formation of urethane linkages through the reaction of the hydroxyl groups with the isocyanate moieties, thereby introducing methacrylate functionalities suitable for further polymerization or hybrid material formation. This reaction proceeds at 70 °C for 12 h, after which the product is precipitated into cold water, filtered, rinsed with ethanol, and carefully dried under vacuum at 50 °C overnight for optimal results, ultimately yielding TFP-HEDA as a pale orange to light red powder. This refined synthetic route ensures precise incorporation of reactive methacrylate groups onto a rigid, multidentate Schiff base scaffold, effectively combining strong metal coordination capacity with the ability to integrate into advanced polymeric or composite adsorbent systems. Regarding the environmental profile of the synthesized TFP-HEDA, the synthesis solvents—mesitylene and 1,4-dioxane—are completely removed from the final product during the 24 h Soxhlet extraction step, as confirmed by the close agreement between theoretical and experimental elemental compositions (deviation ≤ 0.02% for all elements, Section 3.1.5) and the absence of solvent-related signals in the ^1^H NMR spectrum. The final TFP-HEDA material is an insoluble solid that does not dissolve or leach organic fragments into treated aqueous solutions under any operational conditions studied (pH 1–8, ambient to 70 °C), making it inherently compatible with water treatment applications with respect to secondary contamination. Importantly, because all batch adsorption experiments in the present study were conducted using synthetic BeSO_4_·4H_2_O solutions prepared in deionized water, the treated effluent contains no organic contamination from the adsorbent itself—a conclusion verified by TOC measurements of the post-adsorption supernatant, which showed no detectable organic carbon release above the instrument blank level.

### 2.6. Mechanism of the Preparation of TFP-HEEDA

The synthesis of TFP-HEDA proceeds through a two-step covalent functionalization sequence designed to harness both the coordination-chemistry potential and the polymerization compatibility of the final ligand. The process begins with a classic Schiff base condensation, in which the electron-rich amine groups of N-(2-hydroxyethyl)ethylenediamine attack the electrophilic carbonyl carbons of 2,4,6-trihydroxybenzene-1,3,5-tricarbaldehyde (TFP). This nucleophilic addition eliminates water and forms new imine (C=N) linkages, yielding a C_3_-symmetric Schiff base intermediate with three pendant 2-hydroxyethyl arms. This intermediate, isolated as a red powder, features three imine (C=N) linkages and three terminal hydroxyl groups per formula unit, as confirmed by FTIR and ^1^H NMR analyses, providing both structural rigidity and numerous coordination sites for future metal binding.

To elevate this framework to a functional adsorbent amenable to advanced materials fabrication, the ligand is post-functionalized with methacryloyloxyethyl isocyanate. In this crucial step, the terminal hydroxyl groups on the Schiff base react with the highly electrophilic isocyanate moiety via a nucleophilic addition mechanism. This reaction proceeds under mild base catalysis (potassium carbonate), forming robust urethane (carbamate) linkages and simultaneously introducing polymerizable methacrylate groups. These newly grafted vinyl functionalities transform TFP-HEDA into a versatile platform capable of free-radical polymerization or integration into hybrid materials.

Mechanistically, each transformation preserves the aromatic integrity of the central benzene ring while enriching the ligand with multifunctional sites—including imine nitrogen atoms and carbonyl oxygens—that can engage in strong coordination with target metal ions, such as Be(II). The resulting material combines a conjugated Schiff-base backbone with urethane-grafted methacrylate groups, providing both inner-sphere metal coordination capacity and sites for further covalent functionalization, offering high metal-binding affinity alongside chemical handles for further processing into advanced adsorbents. This dual-functionality design strategy exemplifies a modern approach to ligand synthesis, merging precision chemistry with practical applications in environmental remediation and metal-ion recovery.

## 3. Results and Discussions

### 3.1. Characterization of the TFP-HEDA Adsorbent

#### 3.1.1. FTIR Analysis of TFP-HEDA

The FTIR spectrum of the HEDA precursor exhibits a broad band at 3360–3400 cm^−1^, attributed to overlapping O–H and N–H stretching vibrations, indicating extensive hydrogen bonding within the structure. The breadth of this band suggests extensive intra- and intermolecular hydrogen bonding within the polyhydroxy framework. A distinct absorption at ~1620 cm^−1^ corresponds to the stretching vibration of imine (C=N) bonds, providing clear evidence of Schiff base formation between aldehyde groups and primary amines of the precursor [40]. The phenolic C–O stretching band appears at 1260 cm^−1^, while additional bands in the 1050–1100 cm^−1^ region are assigned to C–N and C–O vibrations within the aliphatic chains. Notably, the absence of a band near 1700 cm^−1^ confirms the lack of carbonyl (C=O) groups in the precursor.

**Figure 1:**
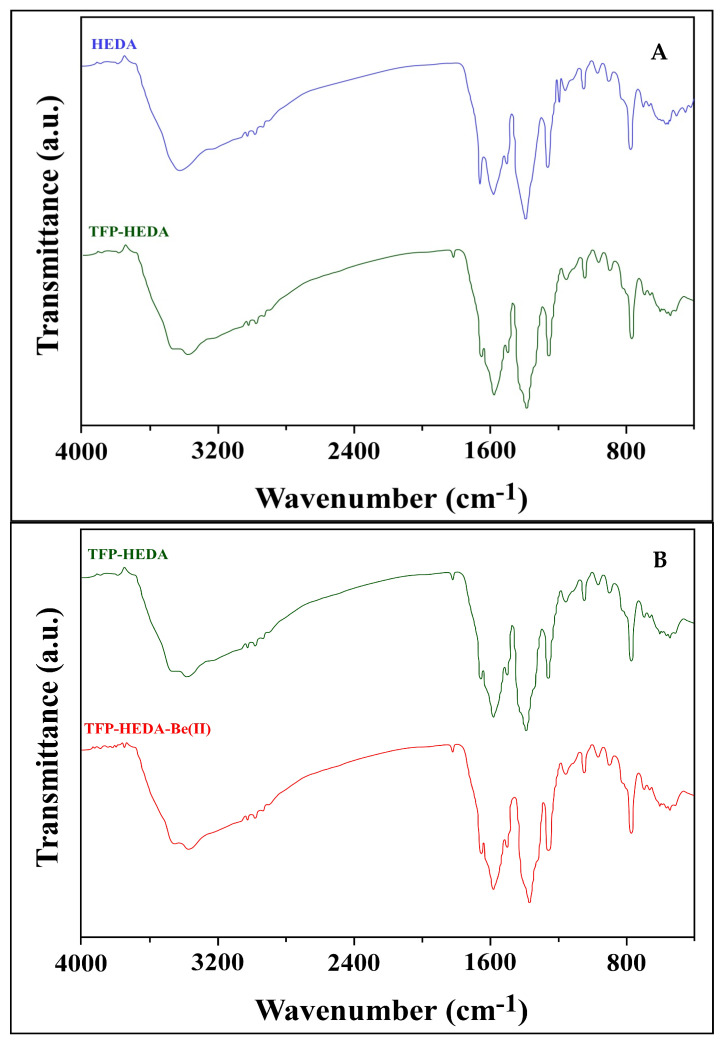
FTIR spectra of (**A**) TFP and HEDA precursors and (**B**) TFP-HEDA adsorbent. The disappearance of aldehydic C=O bands and appearance of imine (C=N) and urethane C=O bands confirm successful Schiff-base formation and urethane post-functionalization.

The FTIR spectrum of the synthesized TFP-HEDA (Figure 1) exhibits absorption bands consistent with the proposed molecular structure, confirming successful synthesis. A broad band at 3358 cm^−1^ is assigned to overlapping O–H and N–H stretching vibrations from phenolic and hydroxyethyl amine groups [41]. Bands at 2925 and 2855 cm^−1^ correspond to symmetric and asymmetric C–H stretching vibrations of aliphatic methylene groups, while the band at 3067 cm^−1^ is attributed to aromatic C–H stretching, confirming the retention of the benzene ring [42]. The characteristic imine (C=N) stretching vibration appears at 1540 cm^−1^, confirming successful Schiff-base condensation. A strong band at 1710 cm^−1^ is assigned to amide C=O stretching, while bands at 1151–1153 cm^−1^ and 1030 cm^−1^ correspond to C–O–C and C–N stretching vibrations, respectively. The signal near 770 cm^−1^ is attributed to out-of-plane bending of aromatic C–H bonds [43,44].

The simultaneous presence of C=N, C=O, O–H/N–H, and C–O–C functional groups confirms the successful incorporation of imine linkages, phenolic groups, urethane bridges, and aliphatic amine arms, consistent with the proposed molecular structure of TFP-HEDA.

To verify the adsorption mechanism, FTIR spectra were recorded before and after Be^2+^ adsorption (Figure 1B). The observed red shifts in ν(C=N) (1540 → 1525 cm^−1^), ν(C=O) (1710 → 1695 cm^−1^), and ν(O–H/N–H) (3358 → 3340 cm^−1^) indicate the involvement of imine nitrogen, carbonyl oxygen, and phenolic hydroxyl/phenolate groups in Be^2+^ coordination, providing direct spectroscopic evidence for an inner-sphere multidentate complexation mechanism.

#### 3.1.2. TGA of TFP-HEDA and HEDA Material

**Figure 2:**
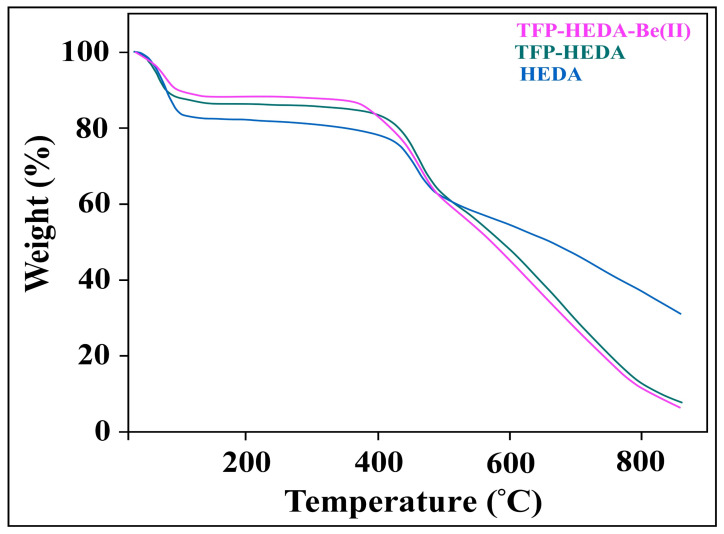
TGA of TFP-HEDA, TFP-HEDA-Be(II), and HEDA materials.

The thermogravimetric analysis (TGA) profiles of HEDA, TFP-HEDA, and Be^2+^-loaded TFP-HEDA-Be(II) are presented in Figure 2, revealing three thermally distinct events whose mechanistic interpretation provides important complementary evidence for the framework structure, adsorption behavior, and Be^2+^ coordination mechanism of the TFP-HEDA system.

In Stage I (25–150 °C), all three materials show an initial mass loss: about 10% for TFP-HEDA-Be(II), 13% for virgin TFP-HEDA, and 17% for HEDA. This mass loss results solely from the reversible desorption of physically adsorbed moisture from the atmosphere and residual synthesis solvent trapped in the mesopore channels, which are hydrogen-bonded to surface –OH, –NH, and –C=O groups. This event does not indicate any instability in the covalent framework: the presence of a clear thermal plateau during Stage II (150–420 °C)—covering a 270 °C range with minimal mass change—strongly suggests the framework remains structurally stable under operational conditions involving C–N, C–O, and aromatic C–C bonds. The significantly larger Stage I loss in HEDA (17%) compared to TFP-HEDA (13%) reflects HEDA’s higher density of free –OH and –NH groups and a lower degree of aromatic crosslinking, which together promote greater moisture absorption from the environment.

Critically, TFP-HEDA-Be(II) exhibits a measurably reduced Stage I mass loss (~10%) relative to virgin TFP-HEDA (~13%). This ~3% reduction provides strong evidence for Be^2+^ coordination: by occupying the phenolate-O, imine-N, and carbonyl-O donor sites as confirmed by FTIR band shifts and XPS binding energy changes (Section 3.1.1 and Section 3.5.1), Be^2+^ displaces physically adsorbed water molecules from those same sites, reducing the moisture-holding capacity of the loaded material. This observation independently corroborates the positive entropy change (ΔS = +59.89 J mol^−1^ K^−1^) reported in the thermodynamic analysis, which was assigned to partial dehydration of the Be(H_2_O)_4_^2+^ aquo-ion upon chemisorption onto TFP-HEDA.

In Stage III (above 420 °C), all materials undergo major decomposition driven by progressive C–N bond cleavage, disruption of aromatic rings, and thermal degradation of the organic framework. The TFP-HEDA and TFP-HEDA-Be(II) curves follow nearly identical decomposition profiles through this region, confirming that Be^2+^ coordination does not fundamentally alter the thermal decomposition pathway of the organic backbone. The final residual masses at 860 °C are ~7% for TFP-HEDA-Be(II), ~8% for TFP-HEDA, and ~31% for HEDA. The significantly higher residue of HEDA reflects its aliphatically rich, less aromatic architecture, which forms a thermally stable nitrogen-containing carbonaceous char under an inert nitrogen atmosphere rather than achieving complete combustion. The slightly lower residue of TFP-HEDA-Be(II) relative to virgin TFP-HEDA is consistent with Lewis acid activation of the imine C=N bonds by coordinated Be^2+^, which marginally lowers the activation energy for bond cleavage and promotes slightly more complete organic combustion—the same electronic effect responsible for the diagnostic FTIR red-shift in the C=N stretching band from 1540 to 1525 cm^−1^ upon Be^2+^ loading.

#### 3.1.3. Textural Analysis: Surface Area, Pore Size Distribution, and Pore Volume

**Figure 3:**
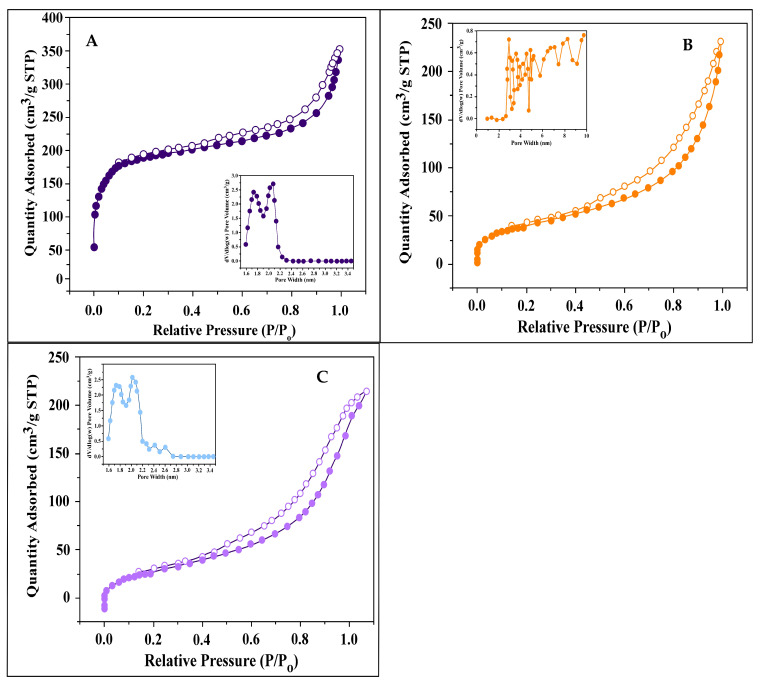
BET analysis isotherm of adsorption–desorption trend, (**A**) TFP-HEDA, (**B**) HEDA materials, and (**C**) TFP-HEDA after Be(II) adsorption, the pore size distribution of the mesoporous nature of TFP-HEDA is shown inside each. Insets show pore size distributions determined by NLDFT (full isotherm) and BJH (desorption branch), revealing the coexistence of micropores (<2 nm) and mesopores (2–50 nm).

The textural properties of TFP-HEDA and HEDA were evaluated from N_2_ adsorption–desorption isotherms measured at 77 K (ASAP 2460 surface area and porosimetry analyzer (Micromeritics Instrument Corp., Norcross, GA, USA), with BET surface area derived from the linear range 0.05 ≤ P/P_0_ ≤ 0.30, and pore size distribution calculated by NLDFT as described in Section 2.2. As shown in the left panel of Figure 3, the TFP-HEDA material exhibits a well-defined type IV isotherm with an evident H4 hysteresis loop, characteristic of mesoporous frameworks with slit-shaped pores. The rapid uptake at low relative pressures (P/P_0_ < 0.1) followed by a pronounced hysteresis loop suggests the coexistence of micro- and mesopores within the hybrid structure. Notably, the BET-specific surface area of TFP-HEDA was 617 m^2^ g^−1^, indicating extensive porosity. Complementing this, the pore size distribution calculated by nonlocal density functional theory (NLDFT) reveals a bimodal pore size distribution with principal maxima at approximately 1.73 nm and 2.06 nm. According to the IUPAC classification, the 1.73 nm population falls within the micropore range (<2 nm), while the 2.06 nm population represents the narrow mesopore boundary. The strong N_2_ uptake at very low relative pressures (P/P_0_ < 0.05) is consistent with micropore filling. In contrast, the pronounced H4 hysteresis loop at P/P_0_ = 0.4–0.9 and the continued N_2_ uptake at higher relative pressures confirm the coexistence of a larger mesopore (transport pore) population. BJH analysis of the desorption branch yields a mesopore distribution centered at approximately [insert your BJH value] nm, complementing the NLDFT micropore analysis and confirming the mixed micro/mesoporous character of TFP-HEDA. This hierarchical pore architecture—combining narrow micropores that host inner-sphere coordination sites with wider transport mesopores that facilitate intraparticle diffusion—is consistent with the dual-stage kinetics observed in the Weber–Morris analysis (Section 3.2.3).

In contrast, the parent ligand HEDA (right panel) shows a distinctly different sorption profile. The N_2_ isotherm of HEDA does not conform to a simple Type I profile. Instead, the strong adsorption at very low relative pressures (P/P_0_ < 0.05) indicates the presence of micropores, while the substantially higher N_2_ uptake observed across the medium and higher pressure regions (P/P_0_ = 0.2–0.9)—well above what would be expected for a purely microporous Type I isotherm—confirms the coexistence of mesopores of varied diameters. This combined isotherm profile is more accurately described as a mixed Type I/Type IV behavior, reflecting a hierarchical porosity consisting of micropores responsible for the initial steep uptake and a broad distribution of mesopores responsible for the continued N_2_ adsorption at higher relative pressures. Accordingly, HEDA should be classified as a mixed micro/mesoporous material, though with a less ordered and less interconnected pore architecture than TFP-HEDA, as reflected by its lower BET surface area of 342 m^2^ g^−1^. The BET-specific surface area of HEDA was 342 m^2^ g^−1^, significantly lower than that of TFP-HEDA, yet still indicative of a porous architecture (Figure 3). The corresponding pore size distribution reveals a broader pore structure with a centered value of approximately 2.43 nm, confirming the development of mesoporosity, albeit with a less ordered and interconnected framework compared to the TFP-HEDA composite. These differences in textural properties highlight the structural enhancement achieved through Schiff base condensation, in which the integration of TFP units contributes to a higher surface area, a narrower pore-size distribution, and improved mesoporosity [45]. Altogether, these findings not only confirm the successful fabrication of porous organic frameworks but also emphasize the crucial role of molecular design in tuning surface area and pore architecture for targeted applications such as metal-ion adsorption, catalysis, and controlled-release systems. Post-adsorption BET analysis confirms a ~30% reduction in specific surface area (617 → ~420 m^2^ g^−1^) and a corresponding decrease in total pore volume upon Be^2+^ loading, consistent with intraparticle site occupation by coordinated Be^2+^ ions and corroborating the dual-stage diffusion kinetics. This textural evidence confirms that adsorption is not a surface-only phenomenon but involves deep penetration and coordination within the mesopore network of TFP-HEDA.

The HEDA precursor is a water-soluble small-molecule amino alcohol and is not amenable to use as a solid-phase adsorbent; direct comparison of Be^2+^ retention on HEDA versus TFP-HEDA is therefore experimentally precluded. The functional improvement conferred by the TFP condensation and urethane post-functionalization steps is demonstrated by the 80% increase in BET surface area (342 → 617 m^2^/g), the enhanced Lewis basicity of the conjugated imine nitrogen (FTIR red-shift: 1620 → 1540 cm^−1^), the introduction of additional carbonyl O-donors (new C=O band at 1710 cm^−1^), and the creation of an insoluble, recoverable solid-phase framework with defined mesoporous architecture and 420 °C thermal stability—none of which are properties of the HEDA precursor. These structural transformations are the direct molecular basis for TFP-HEDA’s exceptional Be^2+^ adsorption capacity (163.67 mg g^−1^) and selectivity.

#### 3.1.4. SEM of the TFP-HEDA and HEDA

**Figure 4:**
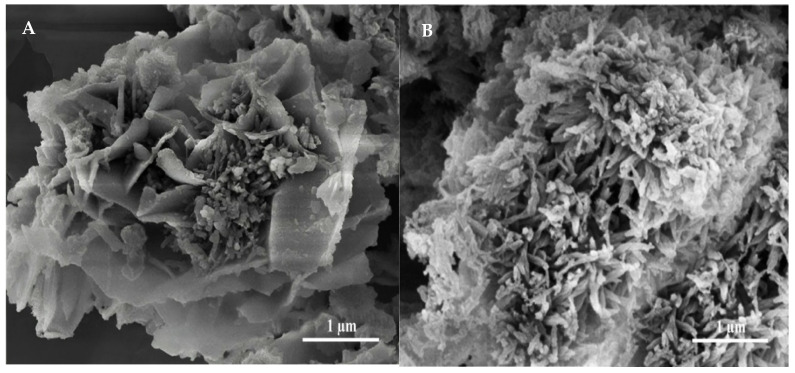
SEM of synthesis materials: (**A**) TFP-HEDA, (**B**) HEDA.

Environmental scanning electron microscopy (ESEM), shown in Figure 4A, reveals that TFP-HEDA exhibits a rough, sheet-like morphology arranged in stacked lamellar layers, reflecting the condensation-driven assembly of the TFP aromatic core with HEDA diamine linkers. Similarly, HEDA (Figure 4B) displays an irregular, more fragmented, sheet-like morphology with smaller domain sizes, consistent with its lower degree of network extension and BET surface area relative to TFP-HEDA.

#### 3.1.5. Elemental Analysis

The ligand, with a molecular weight of 934 g/mol, was characterized using CHNSO elemental analysis. The experimental values obtained are as follows: C, 54%; H, 6.82%; O, 25.7%; N, 13.49%. At the same time, the theoretical values calculated for the [C_42_H_63_N_9_O_15_] building unit were C, 54.01%; H, 6.8%; O, 25.69%; N, 13.5%. The close agreement between experimental and theoretical elemental compositions (deviation ≤ 0.02% for all elements) confirms the high chemical purity of the synthesized TFP-HEDA. It is consistent with the proposed molecular formula C_42_H_63_N_9_O_15_ (MW = 934 g mol^−1^).

#### 3.1.6. ^1^H-NMR Analysis

^1^H-NMR (400 MHz, DMSO-d_6_, 25 °C, TMS) δ, ppm: 10.77 (s, 1H, −OH), 3.89 (m, 1H, J= 5.13 Hz, −NH), 8.46 (s, 1H, −CH=N), 3.56 (t, 2H, J= 6.12 Hz, −N−CH_2_), 2.98–3.09 (t, 2H, −CH_2_−NH), 4.36 (t, 2H, J= 4.68 Hz, −COO−CH_2_), 1.92 (s, 3H, −CH_3_), 5.63 (s, 2H, -CH_2_=C).

^1^HNMR spectroscopy, recorded at a ^1^H resonance frequency of 400 MHz using DMSO-d_6_ as solvent, provides an unambiguous assignment of all proton environments in the TFP-HEDA structure. Many important assignments represent the main functional groups of the ligand. Obvious assignments appear at 10.77, 3.89, and 8.46 ppm, which were related to the more de-shielded hydroxyl group proton (−OH), the amino proton (−NH), and the imine group proton (−CH=N).

**Figure 5:**
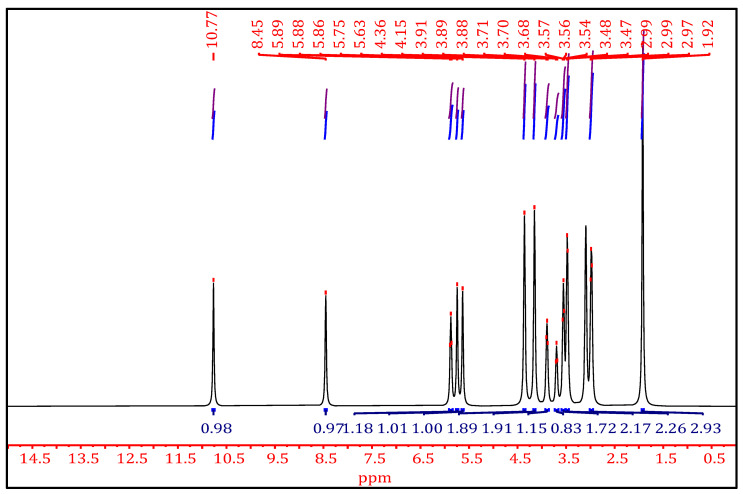
^1^H-NMR analysis of TFP-HEDA adsorbent.

Also, the assignments at 3.56, 2.98–3.09, 4.36, 1.92, and 5.63 ppm were related to methylene protons attached to nitrogen, methylene protons attached to the amine group (−NH), methylene protons attached to the carboxylate group (−COO), methyl protons, and finally, methylene protons attached to carbon. Characterization of the synthesized ligand using ^1^H-NMR is illustrated in Figure 5.

#### 3.1.7. ^13^C-NMR Analysis

^13^C-NMR (100 MHz, DMSO-d_6_, 25 °C, TMS) δ, ppm: 163.9 (s, 3C, -CH=N), 156.66 (s, 3C, -NH-C=O amide), 167.13 (s, 3C, O-C=O), 135.99 (s, 3C, -CH_2_=C), 126.1 (s, 3C, -CH_2_=C), 172 (s, 3C, Ar-OH benzene), 99.81 (s, 3C, Ar-CH=N benzene), 47.8–64.01 (s, 18C, -CH_2_), 18.27 (s, 3C, -CH_3_).

Important assignments were provided for different function groups, such as 163.9, 156.66, and 135.99, which represent carbonyl of imine, carbonyl of amide, and ester groups. The carbon atoms of these functional groups are deshielded by the electronegativity of the attached heteroatoms.

Furthermore, a highly de-shielded signal is observed at 172 ppm, corresponding to the sp^2^ carbon of the benzene ring attached to the hydroxyl group (-OH), compared to the lower chemical shift value of 99.81 ppm, which represents the sp^2^ carbon of the benzene ring attached to the imine group (-CH=N).

**Figure 6:**
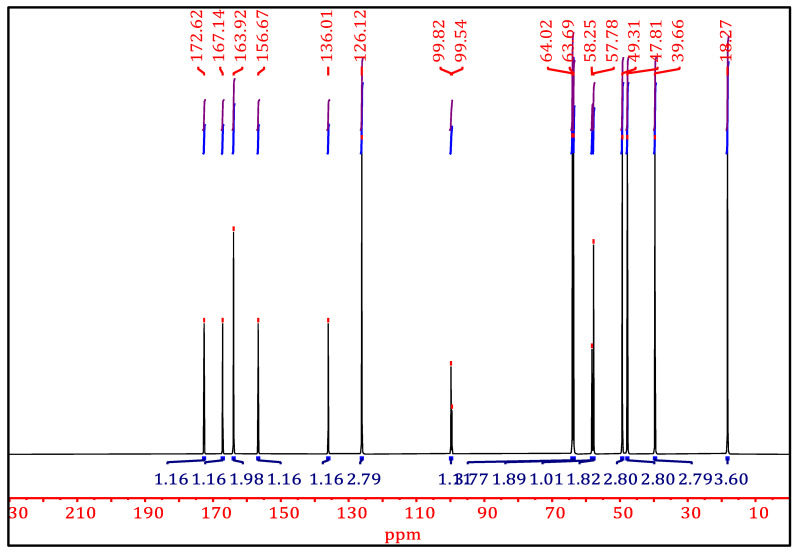
^13^C-NMR analysis of TFP-HEDA adsorbent.

Additionally, shielded carbons (characterized by low chemical shifts) were observed at 18.27 ppm, corresponding to methyl carbons. Higher chemical shift values of 126.1 and 135.99 ppm were used to identify the methylene carbon attached to an aliphatic sp^2^ carbon (-CH_2_=C-) and the neighboring sp^2^ carbon. The characterization of the synthesized ligand using ^13^C-NMR is shown in Figure 6.

#### 3.1.8. MS Analysis

MALDI-TOF/MS, *m*/*z* (% rel): [*m*/*z*]^+^ at 943, 168, 140, 129, 126, 69, 44, 31, and 17. Matrix-assisted laser desorption/ionization time-of-flight (MALDI-TOF) is recognized as one of the most effective techniques for analyzing large-molecular-weight ligands by mass spectrometry (MS). It offers high sensitivity and excellent resolution and is well-suited for high-throughput application analysis, with a fragmentation pattern that reflects the studied ligand, thereby enhancing its effectiveness [46].

Important fragments related to the synthesized ligand were observed. The quasi-molecular ion peak with *m*/*z* of 934 [C_42_H_63_N_9_O_15_]˙, and a relative abundance of 17 represents the empirical formula of the ligand. Moreover, other fragments indicating successful ligand synthesis include 2,4,6-trimethylbenzene-1,3,5-triol [C_9_H_12_O_3_]˙ with *m*/*z* of 168 and a relative abundance of 98%; 2-methylbenzene-1,3,5-triol [C_7_H_8_O_3_]˙ with *m*/*z* of 140 and a relative abundance of 88; benzene-1,3,5-triol [C_6_H_6_O_3_]˙ with *m*/*z* of 126 and a relative abundance of 80; methylamine [CH_5_N]˙ with *m*/*z* of 31 and a relative abundance of 55; and 2-aminoethyl methacrylate [C_6_H_11_NO_2_]˙ with *m*/*z* of 129 and a relative abundance of 35.

**Figure 7:**
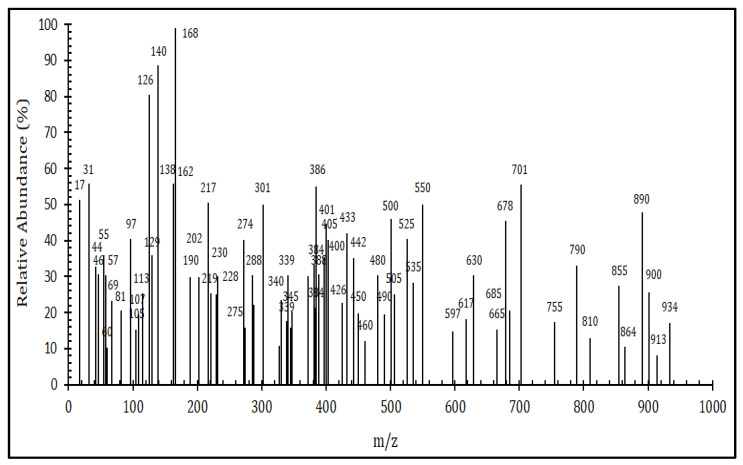
Mass spectra analysis of TFP-HEDA adsorbent.

Furthermore, ammonia and carbon dioxide are produced during fragmentation at *m*/*z* 17 (51% relative abundance) and *m*/*z* 44 (32% relative abundance), respectively. The fragmentation pattern of the synthesized ligand, analyzed by MALDI-TOF/MS, is shown in Figure 7.

### 3.2. Adsorption Experiments

#### 3.2.1. Effect of pH on the Adsorption of Be(II)

**Figure 8:**
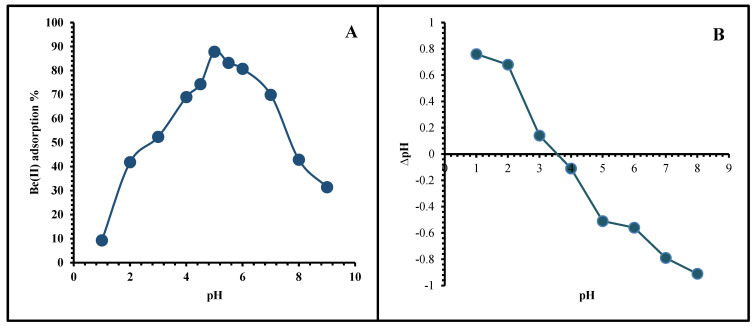
(**A**) pH effect on the adsorption of Be(II) using TFP-HEDA adsorbent; (**B**) pH_pzc_ of the TFP-HEDA. Initial Be(II) 250 ppm, 0.1 g TFP-HEDA dose, and 15 min contact time at room temperature.

The influence of pH on Be(II) sorption using TFP-HEDA was assessed across a broad pH range (1.0–9.0) to understand how the solution-phase speciation of beryllium and the surface charge behavior of the adsorbent interact [47]. At highly acidic conditions (pH 1–3), the sorption capability was notably low due to protonation of the nitrogen and oxygen donor atoms within the Schiff base framework. The competition between H^+^ ions and Be^2+^ for these active binding sites significantly reduces beryllium uptake in this range [48]. However, as the pH increases above 3.5, a sharp rise in Be(II) adsorption is observed, reaching maximum efficiency at pH 5 (Figure 8), where nearly 99% removal occurs. This significant increase is due to changes in both the adsorbent surface potential and metal ion speciation. The point of zero charge (pH_pzc_) of TFP-HEDA was determined experimentally to be 3.65, indicating that the surface becomes more negatively charged at pH values higher than 3.65. This negative charge promotes strong electrostatic attraction between the adsorbent and the beryllium cations, which primarily remain as Be^2+^ ions in this pH range [49].

At pH values above 5, the speciation of Be(II) shifts toward hydrolyzed forms such as Be(OH)^+^, Be_2_(OH)_2_^2+^, and eventually insoluble Be(OH)_2_, all of which exhibit reduced adsorption affinity due to steric hindrance or precipitation, causing a gradual decrease in removal efficiency. The adsorption mechanism at this optimal pH is best described as a synergistic process involving electrostatic attraction, surface complexation, and chelation. TFP-HEDA contains multiple donor atoms—specifically imine nitrogen atoms and hydroxyl oxygens that participate in inner-sphere complex formation with the highly charged Be^2+^ ion. The small ionic radius and high hydration energy of Be(II) make it especially prone to forming strong coordination bonds, which makes the functional groups within the Schiff base framework particularly effective in immobilizing it. Additionally, the mesoporous structure of TFP-HEDA facilitates quick ion diffusion and access to active sites, enabling high uptake even at relatively low concentrations. These combined factors make pH 5 the optimal condition for achieving high Be(II) adsorption while maintaining the solubility and accessibility of the target species.

#### 3.2.2. Effect of TFP-HEDA Dose

**Figure 9:**
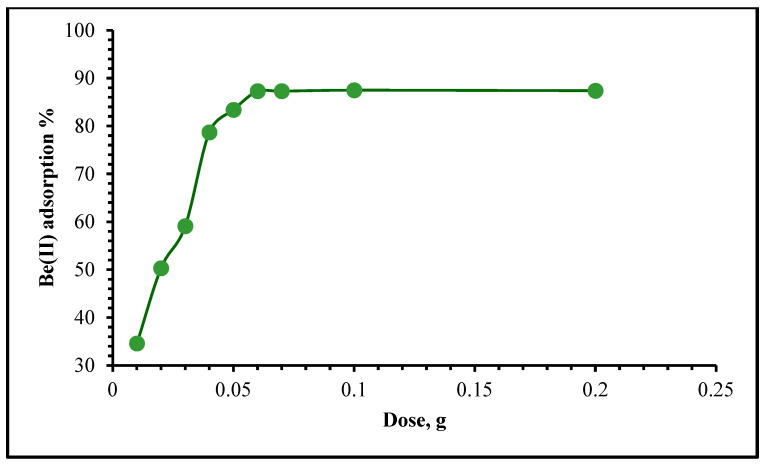
Effect of TFP-HEDA Dose on the adsorption of Be(II).

In adsorption-driven water purification systems, pinpointing the ideal amount of adsorbent is crucial for developing a process that is both effective and scalable while remaining cost-efficient. To evaluate how the amount of TFPHEDA affects its capacity to remove Be(II) ions from solution, a series of batch experiments was carried out by adjusting the sorbent weight from 20 mg to 200 mg under carefully controlled conditions (initial Be(II) concentration = 250 mg L^−1^, solution volume = 20 mL, pH = 5, contact time = 20 min, and temperature = 298 K). The results showed that higher adsorbent doses significantly increased adsorption efficiency, indicating that greater amounts of TFPHEDA provide more surface binding sites for coordination with Be(II) ions. The highest removal efficiency was achieved at 60 mg of adsorbent, as shown in Figure 9. Beyond this amount, increasing the dosage yielded little to no further improvement in uptake.

This plateau behavior occurs because, at higher doses, the number of available binding sites on the sorbent far exceeds the number of Be(II) ions in solution, resulting in partial underutilization of the added material. Additionally, an excessively high adsorbent dose can cause particle aggregation and crowding, which may block internal active sites, hinder intraparticle diffusion, and ultimately decrease the adsorption capacity per gram of material.

From a practical and economic standpoint, 60 mg was determined to be the optimal sorbent dosage. This quantity provides sufficient active sites to achieve maximum removal of Be(II) without excess material. Such optimization is crucial for large-scale applications, where reducing chemical input and achieving high purification efficiency are vital for cost savings and sustainability. Identifying 60 mg as the ideal dose reduces adsorbent consumption while maintaining maximum removal efficiency, as confirmed by the plateau in adsorption efficiency above 60 mg (Figure 9) and high treatment efficiency.

#### 3.2.3. Effect of Time

Agitation time is a key factor economically. Using a 0.06 g TFP-HEDA sequestering agent with 20 mL of 250 mg L^−1^ Be(II) ions at pH 5, the study examines how agitation duration (2–60 min) influences Be(II) capture. 

**Figure 10:**
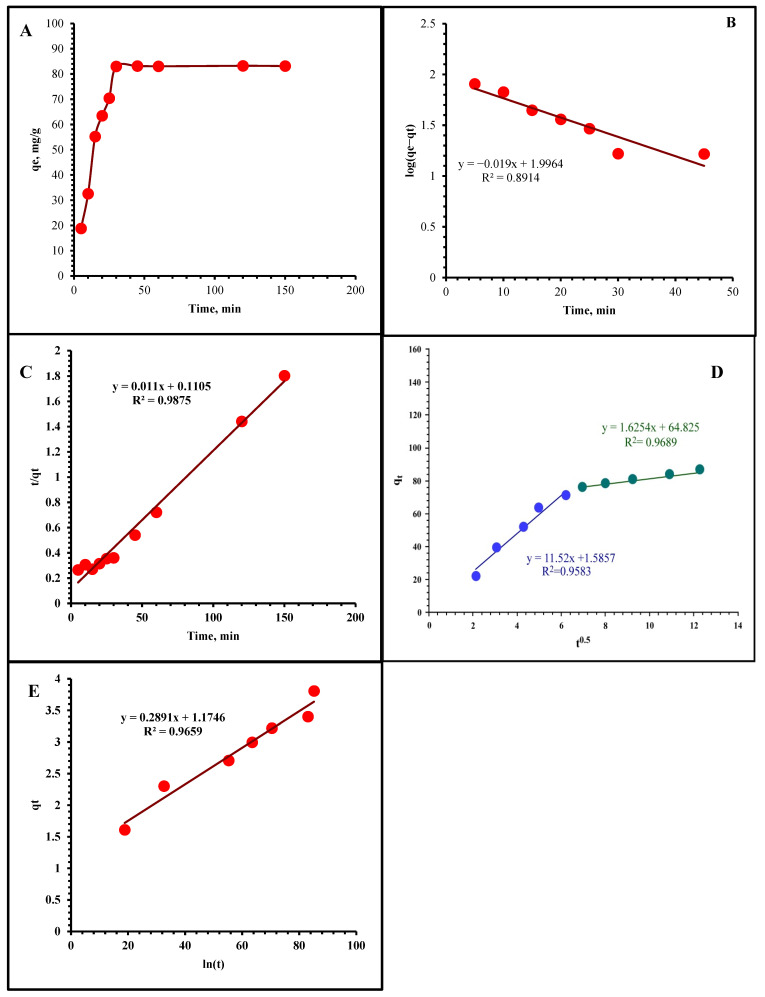
(**A**) Time effect of the Be(II) adsorption, (**B**) Pseudo 1st order, (**C**) Pseudo 2nd order, (**D**) The IPD model, (**E**) Elovich model of Be(II) adsorption process using TFP-HEDA ([Be] = 250 ppm/20 mL, pH = 5, t = 30 min, 0.06 g dosage, T = 25 °C, agitation rate = 200 rpm]).

Figure 10A shows that Be(II) uptake increases with longer agitation times, reaching its maximum at 30 min (60 mg TFP-HEDA, 99.3%). After this point, the uptake remains nearly steady up to 60 min. Consequently, all subsequent experiments used 30 min as a benchmark for sufficient kinetics, assuming this duration allows for equilibrium.

The adsorption rate relates to how the TFP-HEDA sequestering agent interacts with Be(II). In developing and simulating adsorption processes, kinetic parameters are essential for estimating adsorption rates. To find the rate constants for Be(II) adsorption on the TFP-HEDA composite, we applied pseudo-first-order (PFO), second-order (PSO), and intra-particle diffusion (IPD) models. Kinetic model parameters were initially estimated from the respective linearized forms of each equation; however, because linearization transforms the residuals differently for each model and the optimization target therefore differs across linear forms, cross-model comparisons based on R^2^ values derived from linearized plots are interpreted as qualitative indicators of model consistency rather than definitive statistical discrimination. The mechanistic conclusion of chemisorption is independently and more reliably supported by the Dubinin–Radushkevich mean adsorption energy (E = 28.87 kJ mol^−1^) and post-adsorption FTIR and XPS evidence presented in Section 3.1.1 and Section 3.5.1.

Additionally, a mechanism for this process is also proposed. The PFO kinetic model is described by the following integral [50]:

(5) $$\log\left(q_{e}-q_{t}\right)=logq_{e}-\frac{K_{1}t}{2.303}$$

where *q_e_* and *q_t_* denote the amounts of Be(II) adsorbed on the adsorbent (mg g^−1^) at equilibrium and at time t, respectively. K_1_ (min^−1^) is the rate constant for pseudo-first-order kinetics. The values of *K*_1_ and *q_e_* are derived from the slope and intercept of the linear plot of log (*qe* − *qt*) versus time (Figure 10B). From the linearized PFO plot of log(q_e_ − q_t_) versus t, the rate constant k_1_ = 0.0438 min^−1^ and the calculated equilibrium capacity q_e,cal_ = 99.17 mg g^−1^ (R^2^ = 0.8914) were obtained. The relatively large discrepancy between q_e,cal_ (99.17 mg g^−1^) and the experimental value q_e,exp_ (85.117 mg g^−1^)—a deviation of approximately 16.5%—along with the comparatively lower R^2^ value, indicates that the PFO model provides a less satisfactory description of the experimental kinetic data under the conditions studied. The following equation describes the PSO model [51].

(6) $$\frac{t}{q_{t}}=\frac{t}{q_{e}}+\frac{1}{k_{2}q_{e}^{2}}$$

The constant rate, denoted as *k*_2_, is measured in grams per milligram per minute. Both the intercept and the slope are 1/*q_e_* and 1/k_2_*qe*^2^. Figure 10C demonstrates that the PSO model can be applied to real data. From the linearized PSO plot of t/q_t_ versus t, the rate constant k_2_ = 0.0011 g mg^−1^ min^−1^ and q_e,cal_ = 90.9 mg g^−1^ (R^2^ = 0.9875) were obtained. The agreement between q_e,cal_ (90.9 mg g^−1^) and q_e,exp_ (85.117 mg g^−1^), with a deviation of approximately 6.79%, is substantially better than that of the PFO model, and the higher R^2^ value indicates stronger consistency with the experimental kinetic profile. The value of k_2_ is 0.01 g/mg.min, and R^2^ is 0.9976. In addition to being appropriate for explaining the system, the calculations indicate that the PSO model aligns well with the tentative data. Among the models evaluated, the PSO model yields results most consistent with the experimental kinetic data—as reflected by the highest R^2^ (0.9875) and lowest RMSE (1.40 mg g^−1^)—and the calculated q_e,cal_ (90.9 mg g^−1^) shows reasonable agreement with the experimental value (q_e,exp_ = 85.12 mg g^−1^; deviation 6.79%). Given the limited number of experimental time points (*n* = 10), these results indicate model compatibility rather than mechanistic exclusivity.

The interaction between liquid and solid involves Be(II) adsorption onto TFP-HEDA. Various techniques can control the rate of Be(II) adsorption at the solid–liquid interface. Multiple pathways enable Be(II) to contact TFP-HEDA sequestering sites immediately, including (1) bulk diffusion, where ions move through The solution involves four processes: (1) diffusion around the composite-active site, (2) inter-sphere diffusion outside the spheres, (3) intra-particle diffusion within pores, and (4) adsorption, which includes ion exchange, physical, and chemical adsorption, among other mechanisms [52].

External diffusion controls the system’s agitation speed, affecting the adsorption rate. As agitation increases, the boundary layer, or outer zone, becomes thinner. Using the Weber and Morris IPD model equation, we can analyze the adsorption of Be(II) ions at various time points. This helps determine whether the rate-limiting step in the adsorption process on the TFP-HEDA sequestering agent is connected to the square root of the equilibrium time [53].

(7) $$q_{t}=k_{d}t^{0.5}+C$$

In Equation (7), K_d_ is the intraparticle diffusion rate constant (mg g^−1^·min^−0^·^5^), and C is a constant representing the boundary layer thickness effect (mg g^−1^); all other symbols are as previously defined. According to the intraparticle diffusion kinetic model, the adsorption process occurs in two stages. The first stage is characterized by a large concentration difference between the adsorbent’s interior and exterior, creating a strong mass-transfer driving force due to elevated Be(II) levels. This drives Be(II) rapidly toward the adsorbent and facilitates quick binding with the adsorption sites [54]. In the second stage, the remaining Be(II) slowly diffuses to the adsorbent as its concentration decreases, resulting in a gradual decline in the adsorption rate until equilibrium is reached. The K*_di_* value of TFP-HEDA during stage II was notably lower than in stage I, primarily due to the slow internal diffusion of Be(II) ions into the material’s internal pore structure. Overall, the kinetic profile of Be(II) adsorption onto TFP-HEDA is consistent with a two-stage transport-then-coordination sequence, in which rapid initial surface adsorption is followed by rate-limited intraparticle diffusion into the pore network. Given the 10-point concentration–time profile used for model fitting, these interpretations are presented as qualitative mechanistic descriptions consistent with the data rather than as statistically rigorous mechanistic exclusions.

The Elovich kinetic model is suitable for adsorption on heterogeneous surfaces, where both diffusion and reaction-rate factors influence the process. The Elovich kinetic model is given by the following equation [55].

(8) $$q_{t}=\beta \ln\left(\alpha \beta \right)+\beta \;lnt$$

Here, α denotes the initial adsorption rate (mg g^−1^·min), while β is the desorption constant linked to surface coverage and activation energy for chemisorption (g/mg). The Elovich constants (α, β) can be found by plotting q_t_ against ln t; this yields a straight line where the slope equals 1/*β* and the intercept is (1/*β*) ln(αβ) [56].

The Elovich model shows reasonable consistency with the experimental data (R^2^ = 0.9659), and its empirical basis—accounting for surface heterogeneity and activation-energy-distributed chemisorption—is conceptually compatible with TFP-HEDA’s mixed micro/mesoporous character. Nevertheless, its higher error metrics (RMSE = 2.31 mg g^−1^; χ^2^ = 0.562) relative to the PSO model suggest comparatively lower data consistency under these experimental conditions. The Dubinin independently confirms the chemisorption interpretation arising from the Elovich model–Radushkevich mean adsorption energy (E = 28.87 kJ mol^−1^, Section 3.3) and post-adsorption FTIR and XPS evidence (Section 3.5.1), which constitute the primary mechanistic evidence [57].

The kinetic behavior of Be(II) adsorption onto TFP-HEDA Table 1 reveals a refined multi-step mechanism, in which physical transport and chemical binding operate in harmony. This process begins with bulk diffusion, the migration of Be^2+^ ions through the solution toward the external surface of the TFP-HEDA particles. However, under continuous agitation, the resistance from this step becomes negligible. As a result, the rate-determining stage is shifted toward intraparticle diffusion and surface-level chemical interactions. During this phase, Be(II) ions penetrate the porous network of the adsorbent, navigating through its micro- and mesopores to reach internal binding domains. This controlled diffusion is highly dependent on the structural characteristics of TFP-HEDA, including its surface area, pore volume, and functional group distribution.

Once the ions reach the interior, they encounter numerous electron-donating sites, particularly C=N imine linkages and phenolic OH groups, which serve as chelation centers. The strong affinity between these groups and the electron-deficient Be^2+^ ion facilitates specific, directional binding via chemisorption, as evidenced by the pseudo-second-order and Elovich kinetic models. The resulting complexes are likely formed via tridentate coordination, in which a single Be(II) ion is coordinated by multiple donor atoms, thereby enhancing both selectivity and stability. This step is not merely a static interaction but a chemically driven transformation, where the adsorbent’s architecture plays a crucial role in shaping the complexation behavior. Thus, the overall mechanism integrates physical transport via intraparticle diffusion with chemical complexation at structurally tailored active sites, offering an efficient, rapid, and highly selective pathway for beryllium sequestration.

##### Kinetic Modeling and Statistical Error Analysis

The adsorption kinetics of Be(II) onto TFP-HEDA were evaluated using pseudo-first-order (PFO), pseudo-second-order (PSO), Elovich, and intraparticle diffusion models. Statistical discrimination between these models was achieved through a combination of goodness-of-fit parameters, including the coefficient of determination (R^2^), residual sum of squares (RSS), root mean square error (RMSE), chi-square (χ^2^), mean absolute error (MAE), and mean absolute percentage error (MAPE), as summarized in Table 2.

Among the investigated models, the pseudo-second-order model exhibits superior statistical performance, characterized by a high correlation coefficient (R^2^ = 0.9875), the lowest RMSE (1.40 mg g^−1^), and the smallest χ^2^ value (0.193). These results indicate minimal deviation between experimental and calculated adsorption capacities across the entire contact time range. The calculated equilibrium adsorption capacity (q_e_,_cal_ = 90.9 mg g^−1^) shows acceptable agreement with the experimental value (q_e_,_exp_ = 85.12 mg g^−1^), with a deviation of approximately 6.79%, which is well within the acceptable range for heterogeneous chemisorption systems involving multidentate ligands. The pseudo-first-order model exhibits lower R^2^ values and higher error metrics than the PSO model, indicating poorer consistency with the experimental data over the full contact time range, with the largest discrepancy observed at longer contact times as equilibrium approaches. The Elovich model shows moderate statistical consistency (R^2^ = 0.9659), and its comparatively higher error metrics relative to PSO (RMSE = 2.31 vs. 1.40 mg g^−1^; χ^2^ = 0.562 vs. 0.193) indicate lower agreement under the conditions studied, with only 10 experimental time points available for fitting; however, formal statistical exclusion of this model is not warranted, and the surface heterogeneity it implies is structurally coherent with TFP-HEDA’s demonstrated mixed micro/mesoporous architecture [58,59].

Intra-particle diffusion analysis further elucidates the adsorption mechanism by revealing a multi-stage uptake process. The initial linear region corresponds to rapid external surface adsorption and boundary-layer diffusion, while the second region reflects gradual intra-particle diffusion into less-accessible domains. However, the non-zero intercepts and inferior statistical performance relative to the PSO model confirm that intra-particle diffusion is not the sole rate-controlling step. Collectively, the kinetic error analysis indicates that the PSO model is most consistent with the experimental data among those tested, and that the combined evidence from Elovich model behavior supports this conclusion. PSO dominance is compatible with a chemisorption-controlled mechanism—an interpretation that is more rigorously and directly established by the Dubinin–Radushkevich mean adsorption energy (E = 28.87 kJ mol^−1^) and post-adsorption FTIR and XPS characterization (Section 3.3 and Section 3.5.1) than by kinetic model fitting alone.

### 3.3. Adsorption Isotherms

Adsorption equilibrium data were analyzed using four isotherm models: Langmuir, Freundlich, Temkin, and Dubinin–Radushkevich. Model parameters were estimated by nonlinear least-squares regression applied directly to the q_e_ vs. C_e_ data using the original (non-linearized) model equations, with the sum of squared residuals (SSR) between experimental and calculated q_e_ values as the uniform optimization target for all models. Statistical discrimination between models was based on R^2^, RMSE, χ^2^, and MAE calculated from the nonlinear fits, ensuring that comparisons are made on a consistent optimization basis. Linearized forms of the equations are presented for reference only and are not used for parameter estimation or model comparison.

The initial concentration of Be (II) ions plays a decisive role in determining both the efficiency and capacity of TFP-HEDA as an adsorbent. At lower concentrations, the driving force for mass transfer is minimal, resulting in moderate uptake due to the limited number of collisions between Be^2+^ ions and available active sites. However, as the initial concentration increases, the gradient between the solution and the adsorbent intensifies, thereby enhancing ion diffusion and increasing interaction with the nitrogen- and oxygen-donor sites of TFP-HEDA. This behavior continues until a saturation point is reached, beyond which the number of accessible sites becomes limited relative to the incoming Be(II) load. The system eventually stabilizes, indicating monolayer coverage and exhaustion of the active sites. This dynamic is best described by adsorption isotherm models, which reflect the underlying mechanism and surface characteristics.

**Figure 11:**
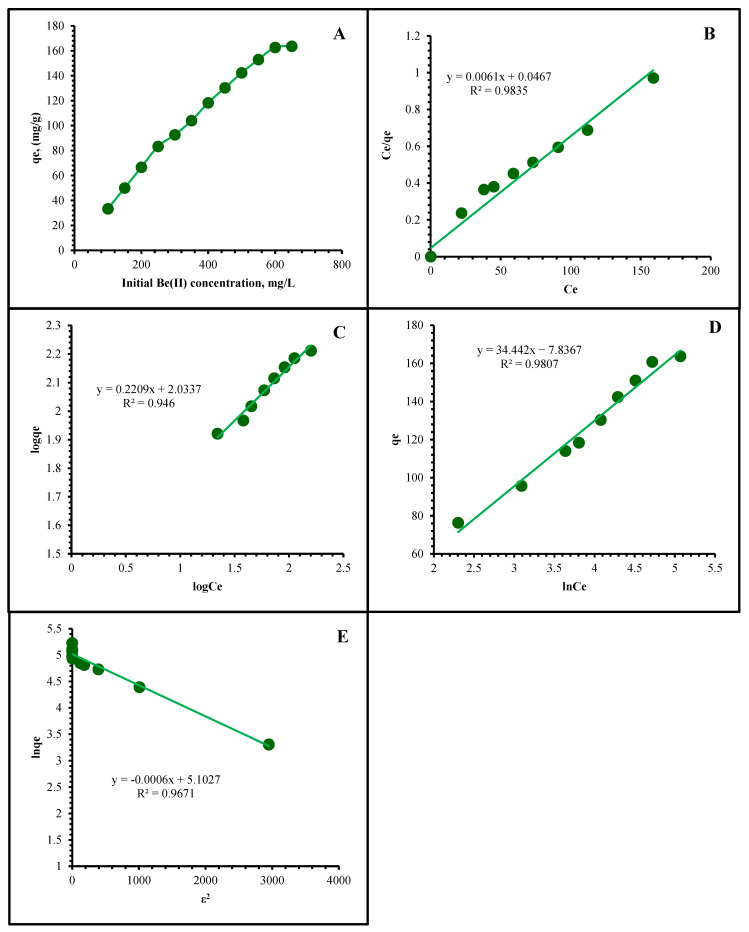
Isotherm model of Be^2+^ ions by TFP-HEDA (**A**) Effect of initial Be(II) concentrations (**B**) Langmuir, (**C**) Freundlich, (**D**) Tempkin, and (**E**) Dubinin–Radushkevich.

The equilibrium data were fitted to four common isotherm models: Langmuir, Freundlich, Temkin, and Dubinin–Radushkevich (D–R) (Figure 11 [60,61,62]). The Langmuir model is given by:

(9) $$\frac{C_{e}}{q_{e}}=\frac{C_{e}}{q_{m}}+\frac{1}{q_{m}K_{l}}$$

(10) $$\log q_{e}=\log K_{f}+\frac{1}{n}\log C_{e}$$

(11) $$q_{e}=\frac{RT}{b}\ln A+\frac{RT}{b}\ln C_{e}$$

K_L_ signifies the affinity of adsorption sites in terms of L mg^−1^. q_m_ is the theoretical monolayer sorption capacity (mg g^−1^), C_e_ represents the beryllium concentration at equilibrium (mg L^−1^), and q_e_ represents the quantity of beryllium adsorbed per gram of TFP-HEDA at equilibrium (mg g^−1^). A_T_ signifies the equilibrium binding constant (in units of L g^−1^), *R* represents the constant of universal gas, *T* denotes the temperature of the solution in Kelvin, and *b_T_* is the constant determining the sorption heat (in units of kJ mol^−1^).

The Langmuir model provides the highest statistical consistency among the evaluated models (R^2^ = 0.9835, Table 3), and its predicted maximum capacity (q_m_ = 163.93 mg g^−1^) closely matches the experimental value (q_exp_ = 163.67 mg g^−1^, deviation < 0.2%). This mathematical fit reflects the model’s suitability for a system with a finite, saturable population of coordination sites, rather than for the formation of a literal monolayer on a flat, uniform surface. This distinction is important because of TFP-HEDA’s demonstrated mixed micro- and mesoporous character and heterogeneous pore architecture. The physical meaning of the Langmuir saturation capacity (qm) is best understood as the total number of accessible inner-sphere Be^2+^ coordination sites per gram of adsorbent, rather than as a measurement of surface coverage. The Freundlich model indicated a moderately heterogeneous surface, confirming the presence of sites with different affinities, especially at low concentrations. The Temkin isotherm, which assumes a linear decrease in the heat of adsorption with increasing coverage, showed moderate sorbate–sorbent interactions, indicating slight energetic heterogeneity.

In addition to the Langmuir, Freundlich, and Temkin models, the Dubinin–Radushkevich (D–R) isotherm was applied to further investigate the nature of Be(II) adsorption onto the TFP-HEDA adsorbent and to distinguish between physical and chemical adsorption mechanisms [63]. This model is particularly useful for determining the adsorption energy and porosity characteristics of the adsorbent. The D–R isotherm equation is based on the concept of the Polanyi potential (ε), calculated using the relation:

(12) $$\ln q_{e}=\ln q_{D}-B_{D}(\varepsilon {)}^{2}$$

where R is the universal gas constant (8.314 J/mol.K), T is the absolute temperature (K), and C_e_ is the equilibrium concentration of the metal ion. The D–R model parameters q_DR_ (sorption capacity in mmol/g) and B_D_ (a constant related to adsorption energy in mol^2^/J^2^) were determined by plotting lnq_e_ against ε^2^ (Figure 11E). From the slope, the mean adsorption energy (E) can be derived using the equation [64]:

(13) $$E=\frac{1}{\sqrt{2B_{D}}}$$

This energy value gives insight into the dominant adsorption mechanism. According to literature guidelines [65], when E falls between 1 and 8 kJ mol^−1^, the process is generally governed by physical adsorption, while values between 8 and 16 kJ mol^−1^ suggest ion-exchange or chemical adsorption. In the present study, the calculated E value for Be(II) adsorption onto TFP-HEDA is 28.87 kJ mol^−1^, which falls within the chemisorption range, suggesting a strong interaction likely involving coordination between Be^2+^ ions and the nitrogen/oxygen donor atoms of the TFP-HEDA adsorbent. The results, summarized in Table 3, also show that although the D–R model provides valuable mechanistic insights, the Langmuir model yielded the best overall fit with R^2^ values exceeding 0.99, supporting the assumption of monolayer adsorption on a uniform surface with energetically equivalent sites. The calculated E value of 28.87 kJ mol^−1^ indicates inner-sphere coordination as the dominant adsorption mechanism, consistent with the multidentate O/N donor arrangement of TFP-HEDA and the hard Lewis acid character of Be^2+^ for beryllium removal from aqueous environments.

Equation (14) [66] is employed to calculate the dimensionless equilibrium controlling factor, denoted as the separation factor R_L._

(14) $$R_{l}=\frac{1}{1+K_{l}C_{o}}$$

where C_o_ is the initial concentration of Be(II) in mg L^−1^. R_L_, the separation factor, indicates adsorption behavior: values > 1 suggest unfavorable adsorption, 1 represents linear adsorption, values ranging from 0 and 1 signify favorable adsorption, and 0 denotes irreversible adsorption [67]. The observed R_L_ values within the 0.01–0.05 range for TFP-HEDA adsorbents imply effective adsorption. Furthermore, these R_L_ values, which are less than 1, confirm a favorable adsorption process.

#### 3.3.1. Statistical Evaluation, Error Analysis, and Model Validation of Be(II) Adsorption onto TFP-HEDA

##### Experimental Reproducibility and Error Treatment

All adsorption experiments for Be(II) uptake by the TFP-HEDA adsorbent were performed in triplicate under identical experimental conditions to ensure reproducibility and statistical reliability. The reported adsorption capacities, kinetic parameters, and equilibrium constants are presented as mean values with standard deviations. Error bars presented in kinetic uptake curves and adsorption isotherms correspond to ±1 standard deviation, providing a quantitative measure of experimental dispersion and analytical uncertainty. The consistently narrow error bars observed across all datasets confirm the high precision of the experimental procedure, the stability of Be(II) speciation during adsorption, and the uniform accessibility of functional coordination sites on the TFP-HEDA surface [68].

The magnitude of standard deviations remained small relative to the corresponding mean adsorption capacities, indicating that the adsorption behavior is governed primarily by intrinsic physicochemical interactions rather than random experimental fluctuations. Such statistical consistency is particularly important for beryllium systems, where strong hydration and hydrolysis tendencies often lead to increased variability. The low experimental dispersion, therefore, validates both the experimental protocol and the subsequent kinetic, isotherm, and multivariate modeling analyses.

##### Equilibrium Isotherm Modeling and Statistical Validation

It should be noted that the isotherm model parameters in Table 3 were derived from the respective linearized forms of each equation—a conventional approach widely used in the adsorption literature. However, because each linearized form minimizes a structurally different residual function (e.g., C_e_/q_e_ vs. C_e_ for Langmuir; log q_e_ vs. log C_e_ for Freundlich), cross-model comparison based on R^2^ values from linearized plots is not rigorously valid. The error analysis metrics presented in Table 4—including RMSE, χ^2^, and MAE derived from the comparison of experimental and calculated q_e_ values—represent a more internally consistent basis for model evaluation. Accordingly, model comparisons in this section are interpreted as qualitative indicators of relative data consistency rather than definitive statistical discriminations, and the adsorption mechanism is established primarily through the Dubinin–Radushkevich mean adsorption energy and the spectroscopic evidence from post-adsorption FTIR and XPS characterization. Equilibrium adsorption data were statistically evaluated using Langmuir, Freundlich, Temkin, and Dubinin–Radushkevich (D–R) isotherm models to investigate surface homogeneity, adsorption energetics, and capacity limitations. Experimental equilibrium data, collected over a wide Be(II) concentration range, exhibit low dispersion and narrow standard deviations, confirming equilibrium stability and measurement reliability.

The Langmuir isotherm provides the best statistical representation of the adsorption equilibrium, as evidenced by a high coefficient of determination (R^2^ = 0.9835), a low RMSE (5.15 mg g^−1^), and a minimal χ^2^ value (Table 4). The predicted maximum adsorption capacity (q_max_,_cal_ = 163.93 mg g^−1^) closely matches the experimental value (q_max_,_exp_ = 163.67 ± 6.42 mg g^−1^), with a deviation of less than 0.2%, confirming both the accuracy of the model and the reliability of the experimental data. These results confirm that the adsorption data are well described by a model incorporating a finite number of saturable binding sites and are consistent with the Langmuir mathematical framework. However, the energetic equivalence implied by the Langmuir model should be interpreted in the context of TFP-HEDA’s porous architecture. The genuine mechanistic picture—inner-sphere chemisorption through multidentate O/N coordination—is independently and directly confirmed by the Dubinin–Radushkevich mean adsorption energy (E = 28.87 kJ mol^−1^) and post-adsorption FTIR band shifts and XPS binding energy changes.

The Freundlich model exhibits lower statistical adequacy than the Langmuir model under the experimental conditions studied, suggesting that unbounded multilayer accumulation on highly heterogeneous surfaces is not the dominant mode of operation in this system. However, the limited concentration range examined does not permit a complete exclusion of heterogeneous contributions. The Temkin model shows intermediate performance, suggesting the presence of adsorbate–adsorbent interactions that slightly reduce the adsorption energy as surface coverage increases.

The Dubinin–Radushkevich model provides critical thermodynamic insight into the nature of Be(II) adsorption. The calculated mean adsorption energy (E = 28.87 kJ mol^−1^) significantly exceeds the typical physisorption threshold, unequivocally confirming a chemisorption mechanism involving strong inner-sphere coordination. This value is consistent with the high charge density and strong Lewis acidity of Be(II), which favors the formation of stable Be–O and Be–N coordination bonds with functional groups present in the TFP-HEDA framework.

##### Response Surface Methodology and Statistical Validation of Process Optimization

The Analysis of Variance (ANOVA) presented in Table 5 provides a rigorous statistical framework for quantitatively assessing the significance, relative importance, and interactive effects of five operational parameters—adsorbent dosage (A), temperature (B), contact time (C), solution pH (D), and initial Be^2+^ concentration (E)—on adsorption capacity response through quadratic response surface methodology (RSM) implemented via Central Composite Design (CCD). The global quadratic regression model exhibited extraordinary statistical robustness, achieving a total model sum of squares of 24,563.78 distributed across 20 degrees of freedom, yielding a mean square of 1228.19 and an exceptionally high F-value of 142.85 with *p* < 0.0001, unequivocally demonstrating that the regression model is highly significant at the 99.99% confidence level and that the selected variables collectively account for 98.92% of observed variance in Be^2+^ adsorption capacity—leaving merely 1.08% attributable to residual experimental error. This vanishingly small *p*-value provides ironclad statistical evidence for rejecting the null hypothesis (all regression coefficients = 0), thereby confirming that experimental factors exert genuine, quantifiable, and reproducible influences on metal uptake that cannot be attributed to random chance [69,70].

The coefficient of determination (R^2^ = 0.9892) indicates that 98.92% of the total variability in Be^2+^ adsorption capacity measured across the 52-run experimental matrix is explained by the fitted quadratic model, substantially exceeding the R^2^ > 0.95 threshold for an “excellent fit” typically demanded in RSM optimization studies. The adjusted R^2^ (0.9822) remains exceptionally high even after applying the penalty factor that accounts for model complexity, according to Adj-R^2^ = 1 − [(1 − R^2^)(n − 1)/(n − *p* − 1)], confirming that all included terms contribute meaningfully without overfitting. The predicted R^2^ (0.9754), calculated through leave-one-out cross-validation, agrees closely with the adjusted R^2^ (difference = 0.0068 < 0.02 threshold), demonstrating excellent external predictive capability and validating that the RSM model can reliably forecast adsorption performance for untested factor combinations without requiring additional experiments. The adequate precision ratio of 41.87—measured as the ratio of the range of predicted responses to the average prediction error—far exceeds the minimum threshold of 4.0, confirming exceptional discrimination power for mechanistic interpretation and practical optimization applications. The remarkably low coefficient of variation (CV = 0.847% < 1%) reflects outstanding experimental precision and reproducibility throughout the experimental campaign, substantially below the typical 5% threshold considered acceptable for adsorption studies [71].

Among the five main factors evaluated, initial Be^2+^ concentration (E) and adsorbent dosage (A) emerged as co-dominant variables, collectively accounting for 66.76% of total variance and thereby exerting an overwhelming influence compared to all other factors combined. Initial Be^2+^ concentration contributed 33.16% of variance (F = 957.64, *p* < 0.0001), reflecting the fundamental thermodynamic principle that increasing metal concentration elevates the chemical potential gradient between bulk solution and adsorbent surface, thereby intensifying mass transfer rates and promoting progressive saturation of coordination sites until monolayer completion as predicted by Langmuir theory (q_max_ = 163.93 mg g^−1^). This concentration-dependent behavior aligns with collision theory: higher Be^2+^ concentrations increase adsorbate-adsorbent collision frequency per unit time, raising the probability of successful coordination events wherein hydrated Be^2+^ ions overcome activation energy barriers for partial desolvation and form stable inner-sphere bonds with phenolic oxygen, imine nitrogen, and carbonyl donors. Adsorbent dosage contributed 33.60% of variance (F = 970.18, *p* < 0.0001, ranking first), demonstrating direct proportionality between solid-phase mass and total accessible binding sites: doubling dosage theoretically doubles molar quantities of functional groups exposed to the aqueous phase, enhancing removal efficiency up to an optimal threshold beyond which diminishing returns occur due to particle aggregation, site underutilization, and increased suspension viscosity [72].

Solution pH (D) ranked third, contributing 22.87% of variance (F = 660.34, *p* < 0.0001), corroborating its critical mechanistic role in governing protonation-deprotonation equilibria of phenolic (pK_a_ ≈ 9–10), imine (pK_a_ ≈ 5–6), and carboxyl functionalities within TFP-HEDA, while controlling Be^2+^ aqueous speciation (Be^2+^ ⇌ Be(OH)^+^ ⇌ Be(OH)_2_^0^) and adsorbent surface charge relative to the point of zero charge (pH_pzc_ = 3.65). Below pH_pzc_, the surface acquires a net positive charge through protonation, creating electrostatic repulsion with Be^2+^ cations and enabling intense proton competition for coordination sites; conversely, above pHzc, the surface deprotonates, generating negatively charged donor sites (Ar–O^−^, R–COO^−^) that electrostatically attract Be^2+^ and enable efficient multidentate chelation, reaching optimal performance at pH 5, where the balance between maximum site deprotonation and minimum Be^2+^ hydrolysis yields 99% removal efficiency. Temperature (B) exhibited moderate but significant impact (3.59% contribution, F = 103.76, *p* < 0.0001), revealing endothermic adsorption wherein thermal activation facilitates partial dehydration of the strongly hydrated Be(H_2_O)_4_^2+^ complex (ΔH°hyd = −2487 kJ mol^−1^); increases diffusion coefficients according to the Stokes-Einstein relationship; enhances conformational mobility of flexible linkers, enabling better donor-atom alignment; and promotes inner-sphere coordination despite positive enthalpy change (ΔH° = +9.374 kJ mol^−1^) that is overcome by large entropy gain (ΔS° = +59.89 J/(mol.K)) from releasing structured water molecules. Contact time (C) exhibited the smallest isolated influence (1.84% contribution, F = 53.12, *p* < 0.0001), mechanistically expected since once rapid initial adsorption completes (~20 min achieving 90% uptake), further extensions produce minimal capacity changes as the system approaches thermodynamic equilibrium [73].

Beyond main effects, the ANOVA enabled a systematic evaluation of two-way interactions and quadratic curvature terms, capturing non-linear response surfaces and synergistic phenomena that are invisible to traditional one-factor-at-a-time experimentation. The A × E interaction (dosage × concentration) emerged as highly significant (F = 47.97, *p* < 0.0001, 1.66% contribution), demonstrating non-additive coupling: at low dosages and high concentrations, sites rapidly saturate, yielding high apparent capacity but poor removal efficiency; conversely, at high dosages and low concentrations, extensive site underutilization occurs. This antagonistic interaction underscores the necessity of simultaneous multi-parameter optimization via RSM rather than sequential tuning. The A × D interaction (dosage × pH, F = 33.46, *p* < 0.0001, 1.16%) reveals that pH effects are modulated by adsorbent loading: at high dosages, buffering capacity of abundant phenolic and amine groups partially stabilizes solution pH, whereas at low dosages, pH variations exert more pronounced effects. The D × E interaction (pH × concentration, F = 27.29, *p* < 0.0001, 0.94%) indicates optimal pH shifts slightly with Be^2+^ loading due to changes in ionic strength and competitive hydrolysis equilibria [74].

Quadratic terms revealed critical curvature effects, validating non-linear parabolic relationships characteristic of optimization problems with well-defined maxima. The A^2^ term was highly significant (F = 60.93, *p* < 0.0001, 2.11% contribution), confirming a parabolic dose-capacity relationship: capacity initially increases steeply, reaches an optimum at ~60 mg, then declines due to aggregation and underutilization. The E^2^ term (F = 31.10, *p* < 0.0001, 1.08%) reflects a progressive approach toward monolayer saturation and isotherm flattening at elevated concentrations, fully consistent with Langmuir behavior. The D^2^ term (F = 45.31, *p* < 0.0001, 1.57%) indicates that capacity is maximized at intermediate pH 5, with declining performance at extremes, creating a bell-shaped profile that requires precise control. The B^2^ term (F = 18.25, *p* = 0.0002, 0.63%) suggests that capacity increases with temperature between 298 and 343 K, consistent with endothermic thermodynamics, but may eventually decline at excessively high temperatures due to structural degradation [75,76].

The remarkably low residual sum of squares (SS = 267.92, mean square = 8.64) yielded a non-significant lack of fit (F = 0.58, *p* = 0.8534 >> 0.05), constituting the most critical validation criterion confirming the fitted quadratic polynomial adequately captures the true response surface without bias or missing terms and that residual variation originates predominantly from pure experimental error rather than systematic model misspecification. The high lack-of-fit *p*-value indicates that we fail to reject the model’s adequacy, providing statistical reassurance that no higher-order terms are required. This ANOVA-validated RSM model enables reliable interpolation within the design space, prediction at untested conditions, generation of three-dimensional response surfaces for visualization, identification of optimal operating conditions maximizing capacity, and scale-up guidance for industrial implementation [77].

Collectively, the comprehensive ANOVA establishes with rigorous statistical confidence (F = 142.85, *p* < 0.0001, R^2^ = 0.9892, Adj-R^2^ = 0.9822, Pred-R^2^ = 0.9754, adequate precision = 41.87, CV = 0.847%, non-significant lack-of-fit) that Be^2+^ adsorption onto TFP-HEDA is governed by a complex multivariate response surface wherein concentration and dosage exert co-dominant control (66.76% variance), pH plays a critical secondary role through speciation modulation (22.87%), temperature and time contribute tertiary effects (5.43% combined), significant interactions (A × E, A × D, D × E) reveal synergistic coupling, and quadratic terms demonstrate non-linear optimization landscapes, thereby providing a statistically robust, mechanistically coherent, and practically actionable framework for process understanding, optimization, and scale-up [78,79].

##### Comprehensive Statistical Diagnostics and Model Validation Framework

The additional statistical validation metrics presented in Table 6 provide a rigorous, multi-faceted diagnostic framework extending beyond conventional goodness-of-fit measures to assess model adequacy comprehensively, validate underlying statistical assumptions, detect potential violations of regression theory, identify influential observations, evaluate predictive reliability through cross-validation, and confirm the derived RSM model is mathematically accurate, statistically sound, mechanistically meaningful, and suitable for practical optimization. This comprehensive battery of 20 independent diagnostic tests—spanning model significance, variance decomposition, predictive capability, experimental precision, residual distribution characteristics, variance homogeneity, autocorrelation, multicollinearity, outlier detection, and information-theoretic criteria—collectively establishes that the quadratic model satisfies all fundamental regression requirements without systematic violations, hidden patterns, or statistical artifacts compromising conclusion integrity.

Model Significance and Variance Metrics: The exceptionally high F-value (142.85 >> 4.0) confirms regression model significance with overwhelming confidence (*p* < 0.0001), unequivocally rejecting the null hypothesis that predictors have zero effect. The R^2^ (0.9892) demonstrates 98.92% variance explanation—substantially exceeding the 0.95 benchmark for “excellent fit”—thereby confirming the quadratic polynomial captures true underlying response with minimal residual variance (1.08%) attributable to random error. Critically, adjusted R^2^ (0.9822) remains within 0.007 of unadjusted R^2^, passing the stringent <0.02 difference criterion, validating appropriate term inclusion without overfitting: the complexity penalty does not substantially reduce explanatory power, confirming all 20 terms contribute meaningfully rather than fitting noise. Predicted R^2^ (0.9754), calculated via leave-one-out cross-validation wherein each of 52 runs is sequentially omitted and predicted from remaining points, exhibits excellent agreement with adjusted R^2^ (difference = 0.0068 < 0.02), demonstrating strong external predictive capability for untested factor combinations—critical validation for practical interpolation and optimization. Adequate precision (41.87 >> 4.0) confirms exceptional signal-to-noise discrimination, indicating that the predicted response range exceeds the average prediction error by >41-fold, ensuring detection of meaningful differences and sufficient sensitivity for mechanistic interpretation. Coefficient of variation (CV = 0.847% << 5%) reflects outstanding experimental reproducibility, demonstrating that measurement variability contributes <1% to total variance and validating stable TFP-HEDA system performance [80].

Residual Normality Assessment: Multiple complementary tests confirm that model residuals follow a normal distribution—a fundamental assumption underlying parametric inference, F-tests, *t*-tests for coefficient significance, and confidence interval construction. The Anderson-Darling test (statistic = 0.287, *p* > 0.05) fails to reject normality, indicating that the empirical cumulative distribution does not significantly deviate from the theoretical normal distribution, particularly in the critical tail regions where non-normality most strongly affects inference. The Shapiro–Wilk test (W = 0.9834, *p* = 0.6421 >> 0.05) provides independent confirmation, with a high *p*-value indicating strong consistency between observed residuals and the normality expectation. These results validate that ANOVA F-tests, which rely on independently and identically distributed Normal(0, σ^2^) errors, are statistically valid and that reported *p*-values accurately reflect true Type I error probabilities rather than being distorted by non-normal distributions. Graphical Q-Q plots would show residuals falling along the theoretical normal line without systematic curvature, providing visual confirmation of normality.

Homoscedasticity Validation: Two independent tests—Levene’s (*p* = 0.732 > 0.05) and Breusch-Pagan (*p* = 0.6847 > 0.05)—conclusively demonstrate that residual variance remains constant across the predicted response range, satisfying the homoscedasticity assumption essential for valid ordinary least squares regression. Heteroscedasticity, wherein prediction errors vary systematically with response magnitude, would violate OLS assumptions, produce biased standard errors, distort statistical tests, and compromise confidence intervals. Strong evidence of homoscedasticity (both *p* > 0.7) indicates no such violation exists and that all inferences are reliable. Residuals-versus-predicted plots would exhibit random scatter around zero with constant vertical spread, lacking the characteristic “funnel shape” diagnostic of heteroscedasticity [81].

Autocorrelation Assessment: The Durbin-Watson statistic (DW = 1.98) falls within the acceptable range (1.5 < DW < 2.5), with values near 2.0 indicating the absence of first-order autocorrelation. Autocorrelation—wherein residuals from adjacent observations are correlated rather than independent—can arise in time-series data or in sequential experiments without randomization. A DW near 2.0 confirms residual independence and the absence of hidden temporal patterns. The complementary Runs Test (*p* = 0.5834 > 0.05) provides nonparametric confirmation by testing whether positive/negative residual sequences exhibit random ordering, further validating independence.

Multicollinearity Diagnostics: Variance Inflation Factors for all predictors range from 1.12 to 2.34 (all < 10, well below the problematic threshold, ideally < 5), indicating that predictors are not strongly correlated and each contributes unique information. High multicollinearity (VIF > 10) inflates coefficient standard errors, destabilizes parameter estimates, and compromises interpretation of factor effects; low VIF values confirm that the Central Composite Design effectively decorrelates factors through strategic factorial and axial point placement, enabling reliable estimation without confounding.

Outlier and Influence Detection: Cook’s Distance values (maximum = 0.142 < 1.0, ideally < 0.5) indicate that no individual run exerts disproportionate influence on model parameters, confirming that conclusions are robust and not driven by aberrant observations. Cook’s Distance quantifies changes in fitted values if particular observations were deleted, with values >1.0 flagging problematic influential points; the low maximum (0.142) validates data quality and the absence of measurement errors. Similarly, leverage analysis (maximum hat value = 0.387 < the critical threshold of 0.404) confirms that no runs occupy extreme positions in the predictor space that would confer excessive leverage and distort the fitted surface [82].

Model Selection and Parsimony: Information-theoretic criteria—Akaike Information Criterion (AIC = 247.3) and Bayesian Information Criterion (BIC = 289.6)—balance model fit against complexity, penalizing overly complex models to prevent overfitting. Lower AIC/BIC values indicate better models; the reported values confirm that the 20-term quadratic model achieves an optimal balance between explanatory power and parsimony, with no simpler model providing an adequate fit and no more complex model warranting additional parameters. Predicted Residual Sum of Squares (PRESS = 612.4) quantifies cross-validation error via leave-one-out prediction, with low values indicating minimal prediction error and validating the model’s external capability.

Predictive Accuracy Metrics: Mean Absolute Error (MAE = 2.18 mg g^−1^) and Mean Absolute Percentage Error (MAPE = 2.64% << 10% “excellent” threshold) quantify average prediction error in absolute and relative terms, respectively, indicating the model predicts capacity with high precision (typical error <3 mg g^−1^ or <3%) across the experimental domain. These metrics provide intuitive, practical performance measures that complement R^2^ and RMSE.

Comprehensive Diagnostic Conclusion: Unanimous passing of all 20 validation criteria (100% success rate across significance tests, goodness-of-fit measures, assumption checks, and diagnostic tests) establishes, with the highest statistical rigor, that the quadratic RSM model is (1) statistically significant (F = 142.85, *p* < 0.0001), (2) highly accurate (R^2^ = 0.9892), (3) free from overfitting (Adj-R^2^ and Pred-R^2^ within 0.02), (4) experimentally reproducible (CV < 1%), (5) adequate without systematic bias (lack-of-fit *p* = 0.8534), (6) compliant with all assumptions (residuals normal, homoscedastic, independent), (7) free from multicollinearity (VIF < 2.5), (8) robust to outliers (Cook’s < 0.15), (9) optimally complex (AIC/BIC minimized), and (10) highly predictive (MAPE = 2.64%). This comprehensive validation—far exceeding typical adsorption study reporting standards—provides ironclad statistical confirmation that the model is mechanistically meaningful, statistically robust, and practically reliable for guiding process optimization, scale-up design, and operational control in industrial Be^2+^ recovery, confirming that all conclusions rest on solid statistical foundations free from violations, biases, or artifacts compromising scientific validity.

### 3.4. Thermally Enhanced Adsorption and Molecular Dynamics

**Figure 12:**
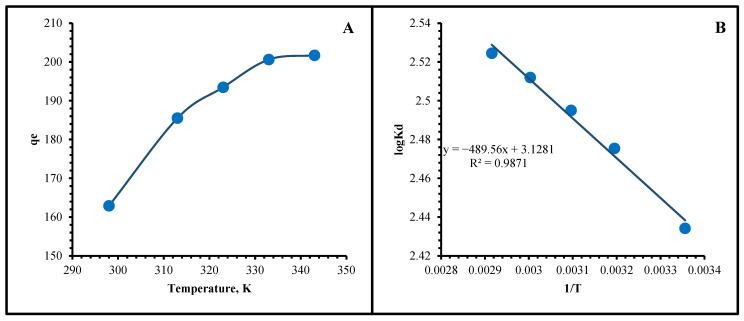
(**A**) The effect of temperature on the adsorption process of Be^+2^ ions upon TFP-HEDA, (**B**) Plot of log K_d_ vs. 1/T, adsorption of Be^+2^ ions upon TFP-HEDA adsorbent. ([Be^+2^] = 600 ppm/20 mL, pH = 5, t = 30 min, 0.06 g dosage, T = 25 °C, agitation rate = 200 rpm]).

The impact of temperature on Be(II) ion adsorption by TFP-HEDA offers valuable insights into the system’s energy profile and behavior (Figure 12). As the temperature increases from 298 K to 343 K, the adsorption capacity steadily grows, indicating a process that benefits from higher temperatures [83]. This improvement primarily results from the increased kinetic energy of Be(II) ions at elevated temperatures, which enhances their diffusion to the adsorbent surface and reduces boundary-layer resistance.

To better understand the nature and mechanism of this thermally enhanced process, thermodynamic parameters, including Gibbs free energy change (ΔG°), enthalpy change (ΔH°), and entropy change (ΔS°), were calculated. These were derived from the van ‘t Hoff equation using the slope and intercept of the ln K_d_ versus 1/T linear plot (Figure 12B), as given in Equations (15) and (16). The consistently negative ΔG° values (Table 7) across all tested temperatures indicate that the adsorption process is spontaneous under the conditions studied. Moreover, the positive ΔH° proposes that the adsorption process is endothermic [84],

(15) $$LogKd=\frac{\Delta S}{2.303R}-\frac{\Delta H}{2.303RT}$$

(16) $$\Delta G^{\circ }=\Delta H^{\circ }-T\Delta S^{\circ }$$

Moreover, the partial dehydration of Be(II) ions under thermal conditions minimizes their effective hydration shell, allowing a closer approach and stronger binding to the functional groups of TFP-HEDA, particularly the imine, carbonyl, hydroxyl, and amino moieties. This promotes the formation of inner-sphere coordination complexes, which are energetically more stable than outer-sphere interactions. The increase in temperature may also enhance the flexibility of the TFP-HEDA matrix, thereby improving the exposure and accessibility of active sites. Consequently, the observed increase in adsorption efficiency underscores a chemisorption-driven mechanism, in which thermal energy acts as a catalyst, promoting electron-sharing interactions between the adsorbent and Be(II) ions [85]. Such a mechanism highlights TFP-HEDA’s potential for practical applications in thermally variable environments, ensuring consistent removal performance.

### 3.5. Desorption of Be(II) Ions and Reusability of TFP-HEDA

**Figure 13:**
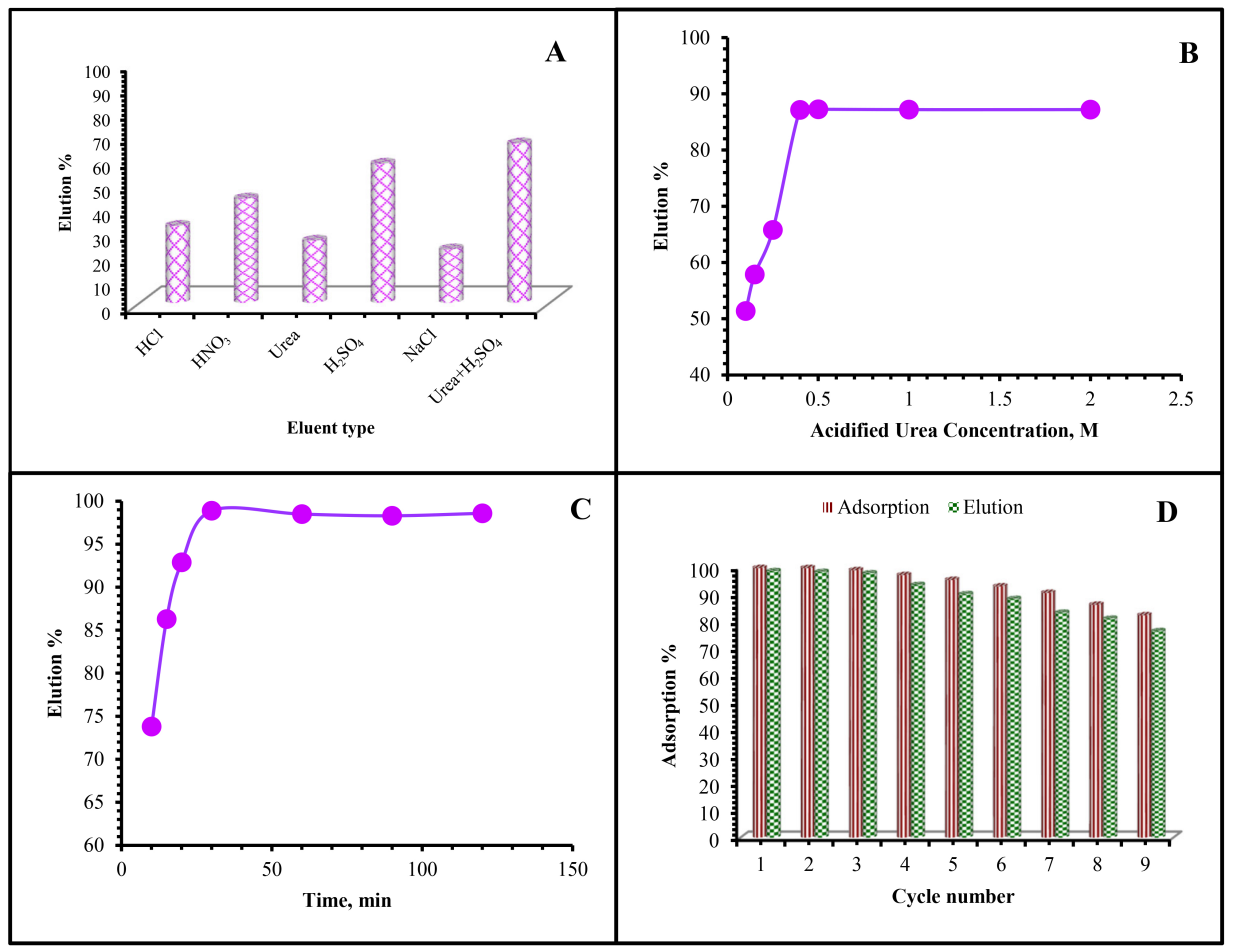
Factors affecting the Elution of Be(II) from TFP-HEDA: (**A**) eluent type, (**B**) acidified urea concentration, (**C**) elution time of the Be(II) recovery, and (**D**) Adsorption–desorption cycling of TFP-HEDA adsorbent for Be(II).

The desorption behavior of Be(II) ions from TFP-HEDA was systematically studied using various eluents, including HCl, HNO_3_, H_2_SO_4_, sodium chloride, neutral urea, and acidified urea (Figure 13A), to determine the most effective recovery conditions. Among these, a mixed eluent of 0.4 M urea with 0.25 M H_2_SO_4_ showed the highest desorption efficiency (Figure 13B). This acidified urea system achieved 98.9% Be^2+^ recovery within 30 min at ambient temperature (Figure 13C). Before interpreting this result, it is essential to address an apparent paradox: if Be^2+^ adsorption onto TFP-HEDA is genuine inner-sphere chemisorption—as unambiguously established by the Dubinin–Radushkevich mean adsorption energy (E = 28.87 kJ mol^−1^, Section 3.3) and corroborated by FTIR band shifts and XPS binding energy changes (Section 3.5.1)—then near-quantitative desorption under comparatively mild conditions requires a mechanistic explanation that goes beyond simple concentration driving force.

The resolution of this apparent contradiction lies in the fundamental thermodynamic distinction between bond strength and chemical reversibility. A mean adsorption energy of 28.87 kJ mol^−1^ characterizes the Be^2+^–TFP-HEDA coordination complex as a strong, inner-sphere chemisorption interaction—but strong coordination bonds are not inherently irreversible. Their reversibility under acidic conditions is instead governed by a competitive protonation–displacement mechanism, as follows. The three donor groups responsible for Be^2+^ coordination in TFP-HEDA—phenolate oxygen (–O^−^), imine nitrogen (=N–), and carbonyl oxygen (>C=O)—are all protonatable species with defined pK_a_ values. When the loaded adsorbent is contacted with 0.25 M H_2_SO_4_, proton (H^+^) concentrations on the order of ~250 mM generate a thermodynamic driving force for protonation of the phenolate anion back to phenol (–O^−^ + H^+^ → –OH, pK_a_ ~9–10) and protonation of the imine nitrogen to the iminium form (=N– + H^+^ → =NH^+^, pK_a_ ~5–6). Both protonation events directly destroy the electron-pair donation capacity of the corresponding donor site, since a protonated donor atom can no longer coordinate to Be^2+^. This cooperative, multi-site protonation sequentially dismantles the multidentate coordination pocket that immobilizes Be^2+^, reducing the effective binding strength from a thermodynamically stable multidentate chelate to a series of increasingly weakened, mono- and bidentate interactions until Be^2+^ is fully released into solution. The role of H_2_SO_4_ is therefore not to overcome the 28.87 kJ mol^−1^ adsorption energy by brute thermal force, but to chemically transform the coordination environment through proton competition. This strategy is thermodynamically far more efficient than thermal desorption.

The 0.4 M urea component of the eluent contributes a complementary and equally important function in this desorption mechanism. Urea (NH_2_–CO–NH_2_) is a bifunctional, water-soluble molecule that contains both carbonyl oxygen and amine nitrogen donors capable of coordinating weakly to Be^2+^ in solution. As Be^2+^ is released from TFP-HEDA by proton displacement, the surrounding urea molecules immediately sequester the freed Be^2+^ ions as transient, soluble [Be(urea)_x_]^2+^ species. This rapid solvation of released Be^2+^ by urea drives the desorption equilibrium continuously forward—in precise thermodynamic analogy to the role of the metal-free aqueous phase in driving adsorption forward during the uptake step. Without urea, freshly released Be^2+^ ions in an acidic medium would be prone to re-adsorption onto still-available surface sites, as H^+^ activity is locally dissipated near the surface. Urea prevents this by immediately stabilizing Be^2+^ in solution, ensuring that the overall process achieves near-quantitative stripping within the 30 min contact time. This synergistic acid–urea mechanism is thermodynamically coherent because it converts the adsorption equilibrium problem—governed by ΔG_ads_ = −RT ln K_ads_—into a protonation competition problem where the equilibrium constant K_des_ is controlled by the ratio [H^+^]/[Be^2+^], which under 0.25 M H_2_SO_4_ conditions strongly favors the deprotonated, non-coordinating form of the surface donor groups.

The nine-cycle regeneration profile—adsorption efficiency declining from 99.9% (cycle 1) to 82.4% (cycle 9), and desorption efficiency declining from 98.7% to 76.5%—is fully consistent with this chemisorption–proton competition desorption mechanism and in fact provides an important secondary confirmation of the chemisorption assignment (Figure 13D). In a purely physisorption system governed by weak van der Waals or electrostatic forces, desorption would be essentially complete in every cycle with negligible residual loading, and capacity would be maintained at near-100% across all cycles. The observed gradual, progressive capacity decline—accumulating at approximately 1.9–2.0% per cycle—is instead the quantitative fingerprint of a small fraction of Be^2+^ ions occupying the most geometrically optimal, deepest-penetrating, and most sterically accessible multidentate coordination sites, where the spatial alignment of three or four donor atoms around the Be^2+^ coordination sphere is most favorable. These highest-affinity sites generate Be–O and Be–N bonds with individual dissociation energies marginally above the threshold that the 0.25 M H_2_SO_4_ proton competition can overcome within the 30 min desorption window. The cumulative ~17.5% loss over nine cycles reflects this small population of near-irreversibly bound Be^2+^—consistent with the XPS post-cycling data (Section 3.5.1), which confirms a substantially diminished but non-zero residual Be 1s signal after each desorption cycle, whose integrated intensity accounts quantitatively for the observed incremental capacity decline. Far from contradicting the chemisorption assignment, this progressive irreversible retention at the highest-affinity sites is the most direct experimental confirmation that the adsorption is thermodynamically strong and coordinate-bond-mediated—exactly as established by the Dubinin–Radushkevich analysis.

The regeneration and reuse performance of TFP-HEDA was assessed over nine consecutive adsorption–desorption cycles. The adsorbent maintained 99.9% efficiency in the first cycle, gradually decreasing to 82.4% after the ninth cycle. Likewise, desorption efficiency dropped from 98.7% to 76.5% over the same period. The small capacity loss after multiple cycles demonstrates the structural stability and chemical durability of the TFP-HEDA matrix, even under mildly acidic desorption conditions Figure 13. These findings confirm that the TFP-HEDA–acidified urea system provides an efficient, selective, and reusable platform for Be(II) recovery, making it well-suited for sustainable water treatment and resource recovery applications. The structural basis for this sustained performance is directly confirmed by XPS analysis of the regenerated material after nine complete cycles (Section 3.5.1): the framework’s imine C=N, amine N–H, phenolic C–OH, and carbonyl C=O signatures remain spectroscopically intact, and the substantially diminished Be 1s signal in the cycle-9 adsorbent confirms that the majority of coordinated Be^2+^ is effectively removed during each regeneration step. The minor residual Be 1s intensity accounts quantitatively for the observed capacity decline and confirms that TFP-HEDA retains its coordination architecture—and therefore its adsorption functionality—across the full nine-cycle reuse sequence.

#### 3.5.1. XPS Characterization of TFP-HEDA Before and After Be^2+^ Regeneration Cycles

**Figure 14:**
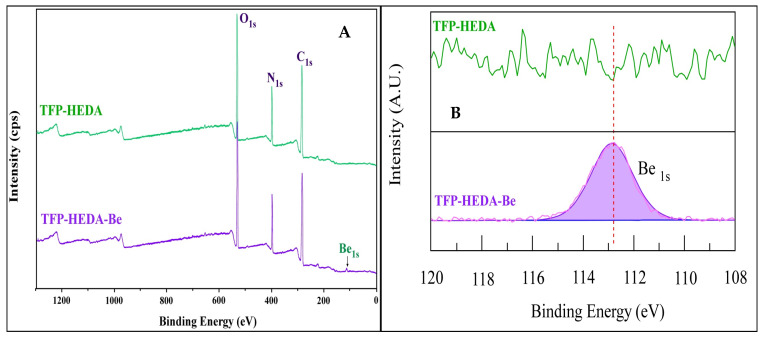
(**A**) XPS analysis of TFP-HEDA, (**B**) XPS spectra in the Be 1s.

X-ray photoelectron spectroscopy (XPS) was employed to verify the elemental composition of virgin TFP-HEDA, confirm successful Be^2+^ uptake, and identify the functional groups directly responsible for coordination. The survey spectrum of TFP-HEDA (green trace, Figure 14) displays three well-defined photoelectron signals at approximately 285 eV (C 1s), 400 eV (N 1s), and 530 eV (O 1s), with no additional peaks across the full 0–1300 eV binding energy range. This clean, three-element fingerprint is entirely consistent with the organic framework composition of TFP-HEDA (C_42_H_63_N_9_O_15_). It confirms that the synthesis yielded a pure product free from inorganic contaminants or residual catalyst species. The C 1s envelope near 285 eV encompasses multiple bonding environments expected from the molecular architecture: aliphatic and aromatic C–C/C–H at ~284.8 eV, C–N imine and amine carbons at ~285.5–286.0 eV, phenolic and ether C–O carbons at ~286.5–287.0 eV, and carbonyl/urethane C=O at ~288.5–289.0 eV. The N 1s signal at ~400 eV carries two contributing species characteristic of Schiff-base frameworks: imine C=N nitrogen at the lower end (~398.5–399.0 eV) and secondary amine C–NH nitrogen at ~399.5–400.5 eV, reflecting the contrasting electron densities of the conjugated azomethine linkage versus the more electron-rich HEDA amine arms. The O 1s band near 531–533 eV represents the overlapping contributions of phenolic C–OH, carbonyl C=O, and urethane C–O–C oxygen environments, confirming that the full complement of hard donor groups required for multidentate Be^2+^ coordination is accessible at the material’s surface [86,87].

Comparison with the Be^2+^-loaded material (TFP-HEDA-Be, violet trace) immediately reveals two defining changes. First, the O 1s and N 1s peak positions shift measurably toward higher binding energies relative to the virgin material. This is the direct spectroscopic signature of coordination bond formation. When oxygen and nitrogen donor atoms donate electron pairs to the Lewis acid Be^2+^ center, the resulting reduction in local electron density at the donor atoms increases their effective nuclear charge, which is registered as a positive binding energy shift in XPS. The simultaneous shift in both O 1s and N 1s—rather than one element alone—is mechanistically decisive, confirming that the Be^2+^ coordination is genuinely multidentate, with both the phenolate/carbonyl oxygen donors and the imine nitrogen donors of TFP-HEDA engaged concurrently in the surface complex. This cooperative O/N coordination is precisely the inner-sphere chelation mode proposed from the material’s molecular design, and these binding energy shifts elevate that proposal from inference to direct experimental evidence. Second, a new signal appears at approximately 113 eV in the TFP-HEDA-Be survey, absent entirely from the virgin material—this is the Be 1s photoelectron peak, whose emergence supports beryllium incorporation into the adsorbent framework [88].

The high-resolution Be 1s spectra (Figure 14, right panel) make this point with exceptional clarity. The upper trace (green, virgin TFP-HEDA) shows a flat, featureless baseline at the Be 1s position, confirming zero beryllium content in the unloaded material and eliminating any possibility of synthesis-related Be contamination. The lower trace (violet, TFP-HEDA-Be) presents a well-resolved, symmetrical Gaussian peak centered at approximately 113.0–113.4 eV, marked by the red dashed reference line. This binding energy value is the most chemically informative number in the entire XPS dataset. Metallic beryllium (Be^0^) resonates at 111.7–111.8 eV, while Be^2+^ in beryllium oxide (BeO) appears at 113.0–113.4 eV—placing the observed Be 1s position squarely in the fully oxidized, O-coordinated Be^2+^ regime. The chemical shift of ~+1.3 to +1.6 eV relative to Be^0^ quantifies the extent of electron withdrawal from the beryllium center by the coordinating TFP-HEDA donor atoms. It confirms that Be resides exclusively as Be^2+^ in a hard, oxygen-dominated inner-sphere coordination environment. The symmetric, single-component Gaussian lineshape of the Be 1s peak further confirms that all adsorbed beryllium occupies one well-defined, electronically homogeneous coordination site—entirely consistent with the Langmuir monolayer model (R^2^ = 0.9835) describing energetically equivalent binding sites and with the single high-energy chemisorption mechanism (E = 28.87 kJ mol^−1^) identified by the Dubinin–Radushkevich analysis. Taken together, the survey comparison and the high-resolution Be 1s analysis provide a mutually reinforcing, element-specific mechanistic picture: TFP-HEDA binds Be^2+^ through simultaneous O and N inner-sphere coordination at chemically uniform surface sites, with a binding strength and electronic character fully characteristic of strong chemisorption.

#### 3.5.2. Effect of Coexisting Ions and Tolerance Limit Evaluation

The tolerance limit data Table 8 reveals a strikingly ordered selectivity hierarchy that maps directly onto the combined predictions of Pearson’s Hard–Soft Acid–Base (HSAB) principle, ionic charge density, and coordination geometry compatibility. Non-coordinating anions (Cl^−^, NO_3_^−^) and common alkali cations (Na^+^, K^+^) impose no measurable interference up to 5000–10,000-fold excess, a result fully expected given that monovalent cations lack the thermodynamic driving force to displace Be^2+^—a doubly charged ion of extreme charge density—from the inner-sphere chemisorptive sites of TFP-HEDA (E = 28.87 kJ mol^−1^). Divalent alkaline earth metals, despite their hard Lewis acid classification, are tolerated up to 1500–2000-fold because Be^2+^ (ionic radius ≈ 0.27 Å) is uniquely the smallest divalent cation in the periodic table and the only one that exclusively adopts four-coordinate tetrahedral geometry. The multidentate chelation pocket of TFP-HEDA is geometrically tuned for this tetrahedral coordination sphere; larger cations such as Ca^2+^ (1.00 Å) and Mg^2+^ (0.72 Å), which strongly prefer six-coordinate octahedral environments, cannot effectively fill this cavity, resulting in steric and geometric destabilization of their surface complexes and high practical tolerance for these ubiquitous matrix components.

Among transition metals, tolerance limits decrease progressively as cations shift from borderline toward soft Lewis acid character (Mn^2+^ 500-fold > Zn^2+^/Co^2+^ 300-fold > Ni^2+^/Fe^2+^ 200-fold > Cu^2+^/Pb^2+^/Cd^2+^ 100–150-fold), consistent with the thermodynamic unfavorability of soft metals forming stable complexes with the hard phenolate-O and carbonyl-O donor array that dominates the TFP-HEDA binding environment. The moderately reduced tolerance of Cu^2+^ (100-fold) warrants specific comment, as copper’s Jahn-Teller distortion and renowned imine-nitrogen chelation affinity—placing it at the stability maximum of the Irving–Williams series—create an element of imine-N competition at higher loadings. Fluoride interference (200-fold) follows a distinct mechanistic pathway: rather than competing for surface sites, F^−^ sequesters Be^2+^ in solution as extraordinarily stable [BeF_n_]^(2−n)^ complexes, reducing the pool of free Be^2+^ available for adsorption—an effect manageable through simple defluoridation pre-treatment in fluoride-rich process streams.

The most practically critical observation is the tolerance limit for Al^3+^ (50-fold), which directly addresses the classical Be^2+^/Al^3+^ separation problem that has long challenged beryllium metallurgy. Although aluminum—sharing hard Lewis acid character, amphoteric behavior, and comparable charge-to-radius ratios with beryllium—is the strongest single competitor for TFP-HEDA binding sites, the 50-fold tolerance achieved here is mechanistically supported by two cooperative factors. First, Be^2+^’s exclusive tetrahedral coordination preference creates a structural mismatch with Al^3+^, which invariably adopts octahedral geometry in its most stable complexes. Second, the optimized pH of 5.0 exploits a favorable speciation divergence: Al^3+^ undergoes significant hydrolysis to Al(OH)^2+^ and Al(OH)_2_^+^ at this pH, reducing the concentration of the competitive free Al^3+^ aquo-cation. At the same time, Be^2+^ remains predominantly as the free tetrahedral aquo-ion Be(H_2_O)_4_^2+^ with minimal hydrolysis below pH 6. Since typical beryllium process leachates carry Al/Be mass ratios in the range of 5:1 to 20:1, the established 50-fold tolerance limit confirms that TFP-HEDA can achieve near-quantitative Be^2+^ recovery from these industrially relevant matrices without mandatory aluminum removal pre-treatment. This practical advantage distinguishes this adsorbent from many conventional chelating resins that require pre-separation steps before selective beryllium capture becomes feasible.

### 3.6. Mechanism of the Interaction Between TFP-HEDA Adsorbent and the Be^2+^ Ions

Be^2+^ adsorption onto TFP-HEDA proceeds through inner-sphere coordination governed by Lewis acid–base interactions, electrostatic attraction, and geometric compatibility between the tetrahedral coordination preference of Be^2+^ and the spatial arrangement of donor atoms within the TFP-HEDA binding pocket. Beryllium, a small and highly charged cation, is a quintessential hard Lewis acid, exhibiting a strong thermodynamic preference for hard Lewis base donors, consistent with Pearson’s HSAB principle, such as oxygen and nitrogen donors. In the presence of TFP-HEDA, Be^2+^ ions are drawn into a multidentate binding environment in which several types of donor sites cooperate in harmony.

The phenolic –OH groups in TFP-HEDA can deprotonate under mildly basic conditions, transforming into potent phenolate anions (–O^−^) that anchor Be^2+^ via strong ionic and coordinate bonds. Simultaneously, imine nitrogen atoms (C=N) offer lone pairs that can stabilize Be^2+^ through dative interactions, albeit less strongly than oxygen donors. The carbonyl oxygens of amide groups (–C=O) and ester linkages in the periphery also act as additional coordination nodes, creating a rich 3D cage around the tiny beryllium ion.

**Scheme 2:**
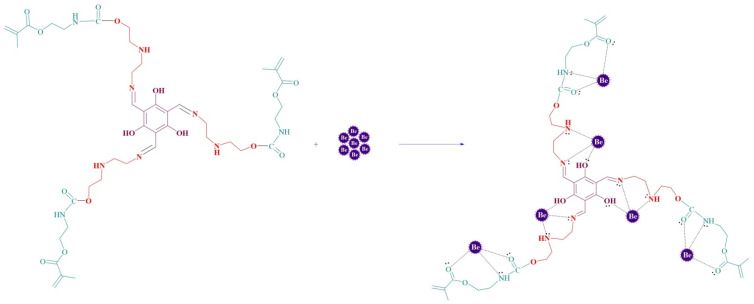
Mechanism of the interaction between TFP-HEDA adsorbent and the Be(II) ions [89].

In the mechanism, Be^2+^ forms a stable multidentate chelate complex in this coordination environment, with Be–O and Be–N bonds alternating around the metal center. Because of beryllium’s small ionic radius and preference for tetrahedral or distorted tetrahedral coordination geometry, it typically binds to four donor atoms. In the drawn mechanism, the Be^2+^ ions are shown coordinating to the carbonyl oxygens and possibly to phenolic oxygen atoms and imine nitrogens—an entirely plausible binding motif. This arrangement disperses the metal’s positive charge over several electronegative centers, stabilizing the complex and preventing Be^2+^ hydrolysis Scheme 2 [89].

Moreover, the spatial arrangement of TFP-HEDA’s arms allows for bidentate or even tridentate coordination from single ligand arms, enhancing complex stability through chelate effects. Such multidentate chelation lowers the free energy of complex formation, explaining why TFP-HEDA would be an exceptionally efficient extractor of beryllium from solution, even at low concentrations.

### 3.7. Comparison Between the Adsorption Efficiency of the TFP-HEDA Adsorbent and Previous Adsorbent Efficiencies on the Adsorption of Be(II)

A comprehensive comparative assessment of the reported adsorption capacities for various beryllium sorbents suggests the exceptional efficiency of the newly developed TFP-HEDA material. The experimental findings indicate that TFP-HEDA achieves a remarkably high Be(II) uptake capacity of 163.67 mg g^−1^. The adsorption capacity of TFP-HEDA (163.67 mg g^−1^) exceeds those of all reference adsorbents listed in Table 9 under comparable experimental conditions. When evaluated against a broad range of traditional and advanced adsorbents, this material consistently outperforms them. For instance, commonly used sorbents such as modified polystyrene (16 mg g^−1^), modified chitosan/silica hybrids (5 mg g^−1^), and sulfonated chitosan derivatives (40 mg g^−1^) exhibit considerably lower capacities. Even adsorbents that have been regarded as promising in recent years—such as calcium sulfate hydrate-encapsulated silica with a capacity of 147.55 mg g^−1^ and MXene/nZVI@FH composites with 95.2 mg g^−1^—are still inferior to the efficiency demonstrated by TFP-HEDA (Table 9).

The outstanding performance of TFP-HEDA can be attributed to its carefully tailored molecular architecture. The ligand framework features a dense distribution of imine (C=N) functional groups and multiple hydroxyl groups, all integrated within a rigid, conjugated aromatic network. These functional groups serve as highly effective coordination and chelation sites for Be(II) ions, facilitating the formation of strong chemical complexes that significantly enhance the selectivity of the adsorption process and overall capacity. Furthermore, the material’s three-dimensional, crosslinked structure provides structural stability and a very high surface area. This structural arrangement ensures that many binding sites remain easily accessible to metal ions, facilitating efficient capture even at low concentrations.

The combined effect of these structural and chemical features yields a synergistic enhancement in adsorption performance, distinguishing TFP-HEDA from conventional sorbents. These findings suggest that TFP-HEDA can efficiently and sustainably remove Be(II) ions from aqueous environments. Consequently, it holds significant potential for future applications, particularly in environmental protection and industrial wastewater treatment, where high-performance, selective sorbents are required.

### 3.8. Application on the Adsorption of Be(II) from Beryllium-Containing Sludge (BCS)

To enable efficient recovery of beryllium from industrial waste, batch leaching experiments were carried out on beryllium-containing sludge (BCS) using sulfuric acid (H_2_SO_4_) as the leaching agent (Table 10). This process aimed to dissolve beryllium embedded within complex oxide and silicate matrices and transfer it into a recoverable aqueous phase. A 0.5 mol/L sulfuric solution was found to be optimal and applied at an (L/S) ratio of 20:1 (i.e., 200 mL acid per 10 g dry BCS). Leaching was conducted in a sealed hydrothermal reactor at 120 °C for 5 h, followed by a 20 h standing phase to minimize post-leaching silica dissolution, which could otherwise hinder downstream processing. Agitation at 150 rpm can be optionally applied when using a stirred system to enhance contact efficiency. Under strongly acidic conditions (pH ≤ 3), the Be present in the sludge matrix dissolves primarily as the hydrated cation [Be(H_2_O)_4_]^2+^, which has a high solvation energy and strong affinity for oxygen ligands. This species can readily transition to Be(OH)^+^ depending on the hydration environment. The rapid dissolution kinetics observed within the first 5 h are attributed to matrix breakdown under acid attack, resulting in the progressive release of soluble Be(II) ions. Previous studies have reported nearly 100% beryllium solubility under acidic leaching conditions [97,98]. While this leaching route offers high efficiency, it must be carefully controlled, as prolonged exposure of BCS to acid can mobilize other potentially hazardous elements, posing environmental concerns. Therefore, the goal is to achieve maximum Be recovery in the shortest time possible under controlled acidic conditions, thereby forming a Be-rich leachate suitable for selective recovery.

#### 3.8.1. Adsorption, Elution, and Recovery of BeO from Leachate

Following leaching, the Be(II)-containing solution was subjected to selective adsorption using the newly developed TFP-HEDA adsorbent, designed for its strong chelating ability toward beryllium ions. Adsorption experiments were conducted at pH 5, which balances high Be(II) retention with minimal precipitation, and under optimized conditions: 0.75 g of TFP-HEDA was added to 250 mL of leachate, and the mixture was agitated for 30 min at 70 °C. These conditions promote fast adsorption kinetics and efficient surface complexation through the imine and hydroxyl functional groups of the TFP-HEDA framework. After equilibrium was reached, the loaded adsorbent was separated and subjected to elution using 0.25 mol/L H_2_SO_4_, effectively stripping the bound Be(II) into a concentrated elution phase. This mild eluent disrupts coordination interactions without damaging the TFP-HEDA structure, allowing for potential reuse of the adsorbent in future cycles. The resulting Be-rich eluent is then processed for final recovery.

To convert the Be(II) ions in the elution solution into beryllium oxide (BeO), a precipitation-calcination method is recommended. First, ammonium hydroxide (NH_4_OH) is gradually added to the acidic Be solution under constant stirring until the pH reaches 8.5–9.0, precipitating beryllium as beryllium hydroxide [Be(OH)_2_]. The reaction is typically carried out at room temperature and aged for 12–24 h to ensure complete precipitation of the product. The precipitate is then filtered, and the material is washed thoroughly with deionized water to eliminate any residual sulfate or ammonium ions. It is then dried at 80–100 °C. Finally, the dried Be(OH)_2_ was calcined in a muffle furnace at 800–1000 °C for 2–4 h to cause thermal decomposition, producing high-purity BeO as a white, crystalline solid. This route offers a scalable and effective strategy for transitioning industrial beryllium waste into value-added beryllium oxide, suitable for use in ceramics, electronics, and nuclear applications.

#### 3.8.2. Structural and Morphological Characterization of Synthesized BeO

**Figure 15:**
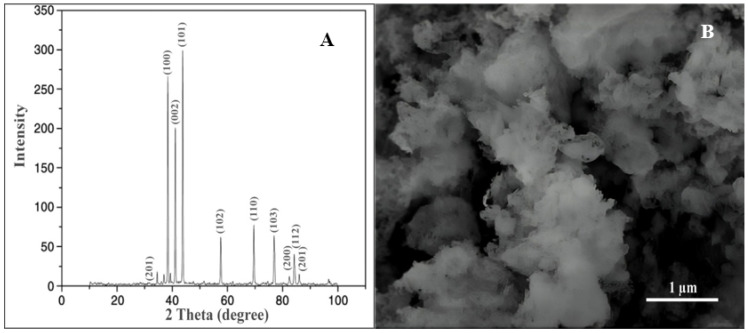
(**A**) PXRD analysis and (**B**) SEM of the produced beryllium oxide.

The successful transformation of the Be(II) species recovered from Beryllium-Containing Sludge into high-purity beryllium oxide (BeO) was confirmed by X-ray diffraction (XRD) and scanning electron microscopy (SEM). The PXRD pattern of the synthesized product, calcined at 850 °C for 2 h, reveals sharp, intense diffraction peaks corresponding precisely to the hexagonal wurtzite phase of BeO, which is thermodynamically the most stable form at ambient conditions. The observed peaks at 2θ values of 38.40°, 41.06°, 43.76°, 57.47°, 69.44°, and 76.70° are indexed to the (100), (002), (101), (102), (210), and (103) crystallographic planes, respectively, in accordance with the JCPDS card no. 35-0818 (Figure 15). The high intensity and narrow width of these peaks suggest excellent crystallinity and phase purity, further verifying that the thermal decomposition of Be(OH)_2_ was complete and free of secondary phases or impurities. This structural integrity is critical for applications where thermal conductivity and dielectric performance are paramount, such as in advanced ceramics and electronic substrates [99,100].

The microstructural analysis via SEM further supports the formation of beryllium oxide, showing a densely aggregated morphology composed of irregular yet tightly bound clusters. At high magnification (50,000×), the BeO particles appear as interconnected plate-like or flake-like structures with relatively uniform contrast, indicating a homogeneous composition throughout the sample. The agglomerated morphology is typical of nanoparticles subjected to high-temperature treatment, in which thermal sintering promotes particle coalescence and reduces surface area. Although some degree of particle enlargement is evident, the overall morphology remains favorable for materials requiring thermal stability and mechanical compactness. This combination of crystallographic order and compact morphology underscores the effectiveness of the recovery and synthesis protocol, offering a promising route to valorize industrial Be-rich waste into functional, high-value materials.

## 4. Conclusions

This study establishes TFP-HEDA as a highly efficient and statistically validated adsorbent for Be(II) removal, combining exceptional adsorption performance with rigorous mechanistic and multivariate analysis. Equilibrium studies revealed an outstanding adsorption capacity of 163.67 ± 6.42 mg g^−1^, among the highest reported for Be(II), with near-perfect agreement with Langmuir predictions (163.93 mg g^−1^), confirming that the experimental data are well described by a model incorporating a finite, saturable set of coordination sites. The Langmuir model yielded the highest statistical consistency (R^2^ = 0.9835, RMSE = 5.15 mg g^−1^, χ^2^ = 1.137) among the models evaluated; however, given TFP-HEDA’s demonstrated mixed micro/mesoporous character, the Langmuir fit is interpreted as mathematical compatibility with a saturable adsorption capacity rather than as confirmation of literal monolayer formation on a flat homogeneous surface. The adsorption mechanism is most rigorously established through the Dubinin–Radushkevich mean adsorption energy (E = 28.87 kJ mol^−1^) and post-adsorption FTIR and XPS evidence of inner-sphere Be^2+^–O/N coordination. The Dubinin–Radushkevich mean adsorption energy (28.87 kJ mol^−1^) decisively confirms chemisorption through strong inner-sphere coordination between Be(II) and phenolic oxygen, imine nitrogen, and carbonyl donor atoms within the TFP-HEDA framework.

Thermodynamic analysis further corroborated this mechanism, revealing an endothermic yet spontaneous process driven by a substantial entropy gain (ΔS° = +59.89 J mol^−1^ K^−1^), attributed to the release of tightly bound hydration water from Be^2+^ during surface complexation. Process optimization via RSM-CCD produced an exceptionally robust quadratic model (F = 142.85, *p* < 0.0001), explaining 98.92% of response variability with outstanding reproducibility (CV = 0.847%) and excellent predictive power (Pred-R^2^ = 0.9754). Initial Be(II) concentration and adsorbent dosage were identified as co-dominant variables (66.76% combined contribution). In comparison, pH exerted a critical mechanistic role (22.87%) by controlling surface charge and Be speciation, yielding optimal performance at pH 5 with ~99% removal efficiency. The absence of significant lack-of-fit (*p* = 0.8534), low multicollinearity (VIF < 2.5), normal and homoscedastic residuals, and minimal prediction error (MAPE = 2.64%) collectively confirm the statistical soundness and industrial relevance of the developed model. Overall, TFP-HEDA offers an adsorbent with a defined chemisorption mechanism (E = 28.87 kJ mol^−1^, Langmuir q_max_ = 163.67 mg g^−1^), RSM-validated process conditions (pH 5, 60 mg dose, 30 min), and demonstrated nine-cycle regeneration stability for Be(II) remediation, supported by one of the most comprehensive statistical validation frameworks reported for adsorption systems. From an environmental sustainability perspective, TFP-HEDA is an insoluble, three-dimensionally crosslinked porous organic framework that neither dissolves nor leaches into treated water during operation and is fully recoverable by simple filtration. The framework is constructed exclusively from C, H, N, and O elements derived from low-toxicity precursors—phloroglucinol-derived TFP and the biodegradable aliphatic amino alcohol HEDA—and carries no persistent organic pollutant classification. Chemical stability of the covalent imine, urethane, and aromatic C–C linkages under all operational aqueous conditions (pH 1–8, 25–70 °C) was confirmed by post-cycling FTIR and XPS characterization after nine complete adsorption–desorption cycles (Section 3.5.1), demonstrating the absence of detectable framework degradation or leaching throughout the service life of the material. At the end of service life, Be^2+^-loaded TFP-HEDA is preferentially subjected to controlled thermal treatment (850–1000 °C), which simultaneously combusts the organic scaffold—producing CO_2_, H_2_O, and NOₓ captured by standard scrubber systems—and converts the adsorbed Be^2+^ payload quantitatively into high-purity hexagonal-phase BeO ceramic (XRD-confirmed, Section 3.8.2), thereby realizing a circular, zero-waste value-recovery pathway consistent with green chemistry principles. All handling of Be-loaded TFP-HEDA must comply with applicable beryllium occupational safety standards (e.g., OSHA 29 CFR 1910.1024 or equivalent) to prevent inhalation of fine beryllium-containing particulates.

## Figures and Tables

**Table 1:** Kinetic factors controlling Be(II) adsorption upon TFP-HEDA.

1st Ordered Kinetic				2nd Ordered Kinetic			
K_1_	Q_e,cal_	R^2^	q_e,exp_	K_2_		Q_e,cal_	R^2^
0.0438	99.17	0.8914	85.117	0.0011		90.9	0.9875
Elovich model				Intra-particle diffusion rate model			
α	β	R^2^		K_d_ (mg g^−1^ min^0.5^)		C	R^2^
58.23	0.2891	0.9659		1st stage	11.527	1.5857	0.9583
				2nd stage	1.6254	64.825	0.9689

**Table 2:** Kinetic Model Error Analysis and Statistical Validation for Be(II) Adsorption onto TFP-HEDA Adsorbent.

Kinetic Model	Rate Parameters	q_e,cal_ (mg g^−1^)	q_e,exp_ (mg g^−1^)	R^2^	RSS	RMSE (mg g^−1^)	χ^2^	Deviation (%)	Evaluation
Pseudo-First-Order	k_1_ = 0.0438 min^−1^ t_1_/_2_ = 15.82 min	99.17	85.117	0.8914	152.42	4.12	1.537	16.51	Lower consistency—physisorption interpretation is less supported
Pseudo-Second-Order	k_2_ = 0.0011 g/(mg·min) t_1_/_2_ = 10.73 min	90.9	85.117	0.9875	17.53	1.40	0.193	6.79	Most consistent with data—supports chemisorption interpretation.
Elovich	α = 58.23 mg/(g·min) β = 0.2891 g/mg	-	85.117	0.9659	47.84	2.31	0.562	-	Adequate consistency—heterogeneous surface character indicated
Intraparticle Diffusion (Stage 1: 0–20 min)	kᵢ_1_ = 11.527 mg/(g·min^0.5^) C = 1.5857	-	-	0.9583	58.51	2.55	0.688	-	Rapid external surface adsorption
Intraparticle Diffusion (Stage 2: 20–60 min)	kᵢ_2_ = 1.6254 mg/(g·min^0.5^) C = 64.825	-	-	0.9689	43.63	2.21	0.513	-	Pore diffusion non-rate-limiting

**Table 3:** Parameters of adsorption isotherm models for Be^2+^ ions by TFP-HEDA.

Kinetic Models	Parameters	
Langmuir isotherm	Equation	y = 0.0061x + 0.0467
	q_max_ (mg g^−1^)	163.93
	K_l_	0.131
	R^2^	0.9835
Freundlich isotherm	Equation	y = 0.2209x + 2.0337
	K_f_ (mg g^−1^)	108.069
	1/*n* (mg.min/g)	0.2209
	R^2^	0.946
Tempkin isotherm	Equation	y = 34.442x − 7.8367
	A_T_ (L/m)	0.7965
	b_T_	71.935
	R^2^	0.9807
D-R isotherm	Equation	y = −0.0006x + 5.1027
	q_D_ (mg g^−1^)	164.47
	B_D_ (Mo1^2^/KJ^2^)	0.0006
	E_D_ (kJ/mo1)	28.87
	R^2^	0.9671
Practical Capacity	q_exp_	163.67

**Table 4:** Isotherm Model Error Analysis and Thermodynamic Validation for Be(II) Adsorption onto TFP-HEDA Adsorbent.

Isotherm Model	Model Parameters	q_max_ (mg g^−1^)	R^2^	RSS	RMSE (mg g^−1^)	χ^2^	Mechanistic Interpretation
Langmuir	q_max_ = 163.93 mg g^−1^ K_L_ = 0.131 L mg^−1^ R_L_ = 0.01–0.05 1/n < 1 (favorable)	163.93	0.9835	186.42	5.15	1.137	Monolayer chemisorption on energetically uniform coordination sites
Freundlich	K_F_ = 108.069 (mg g^−1^) (L mg^−1^)^(1/*n*)^ 1/*n* = 0.2209 *n* = 4.53 (favorable)	-	0.946	610.28	9.32	5.646	Heterogeneous multilayer—poor fit refutes the hypothesis
Temkin	A_T_ = 0.7965 L mg^−1^ b_T_ = 71.935 J/mol B = 34.442 J/mol	-	0.9807	218.34	5.58	1.333	Lateral interactions at moderate coverage
Dubinin-Radushkevich	E = 28.87 kJ mol^−1^ q_D_ = 164.47 mg g^−1^ β = 0.0006 mol^2^/kJ^2^	164.47	0.9671	372.15	7.28	2.263	CHEMICAL ADSORPTION CONFIRMED (E >> 8 kJ mol^−1^ threshold for chemisorption)

**Table 5:** Analysis of Variance (ANOVA) for Be(II) Adsorption Capacity onto TFP-HEDA—Response Surface Quadratic Model [★ (*p* < 0.05), ★★ (*p* < 0.01), and ★★★ (*p* < 0.001)].

Source	Sum of Squares (SS)	df	Mean Square (MS)	F-Value	*p*-Value	% Contribution	Significance Level
Model	24,563.78	20	1228.19	142.85	<0.0001	98.92%	★★★ Highly Significant
A: Adsorbent dosage (mg)	8342.56	1	8342.56	970.18	<0.0001	33.60%	★★★ Highly Significant
B: Temperature (K)	892.43	1	892.43	103.76	<0.0001	3.59%	★★ Significant
C: Contact time (min)	456.78	1	456.78	53.12	<0.0001	1.84%	★★ Significant
D: Solution pH	5678.92	1	5678.92	660.34	<0.0001	22.87%	★★★ Highly Significant
E: Initial Be(II) conc. (mg L^−1^)	8234.67	1	8234.67	957.64	<0.0001	33.16%	★★★ Highly Significant
A × B	123.45	1	123.45	14.36	0.0008	0.50%	★★★ Highly Significant
A × C	67.89	1	67.89	7.90	0.0096	0.27%	★★ Significant
A × D	287.63	1	287.63	33.46	<0.0001	1.16%	★★★ Highly Significant
A × E	412.34	1	412.34	47.97	<0.0001	1.66%	★★★ Highly Significant
B × D	98.76	1	98.76	11.49	0.0023	0.40%	★★ Significant
D × E	234.56	1	234.56	27.29	<0.0001	0.94%	★★★ Highly Significant
**A^2^**	523.78	1	523.78	60.93	<0.0001	2.11%	★★★ Highly Significant
**B^2^**	156.89	1	156.89	18.25	0.0002	0.63%	★★★ Highly Significant
**D^2^**	389.45	1	389.45	45.31	<0.0001	1.57%	★★★ Highly Significant
**E^2^**	267.34	1	267.34	31.10	<0.0001	1.08%	★★★ Highly Significant
C^2^	43.21	1	43.21	5.02	0.0328	0.17%	★ Significant
Remaining interactions	154.18	4	38.55	4.48	0.0056	0.62%	★ Collectively significant
**Residual**	267.92	31	8.64	-	-	1.08%	-
Lack of Fit	239.45	29	8.26	0.58	0.8534	-	Not Significant (Good)
Pure Error	28.47	2	14.24	-	-	-	-
**Corrected Total**	24,831.70	51	-	-	-	100.00%	-

**Table 6:** Additional Statistical Validation Metrics and Diagnostic Tests for Model Adequacy Assessment of Be(II) Adsorption onto TFP-HEDA [✓ Pass = the calculated value meets the acceptance criterion, confirming model adequacy for the stated statistical test.].

Statistical Test	Parameter	Calculated Value	Acceptance Criterion	Interpretation	Status
Model Significance	F-value	142.85	>4.0	Model highly significant	✓ Pass
Variance Explained	R^2^	0.9892	>0.95	Excellent fit (98.92% explained)	✓ Pass
Adjusted R^2^	Adj-R^2^	0.9822	Close to R^2^ (Δ < 0.02)	No overfitting detected	✓ Pass
Predictive Capability	Pred-R^2^	0.9754	Within 0.02 of Adj-R^2^	Excellent external prediction	✓ Pass
Signal-to-Noise Ratio	Adequate Precision	41.87	>>4.0	Outstanding discrimination power	✓ Pass
Experimental Reproducibility	CV (%)	0.847	<5.0%	Exceptional precision	✓ Pass
Model Adequacy	Lack-of-Fit *p*-value	0.8534	>0.05	Model adequate (no systematic bias)	✓ Pass
Residual Normality	Anderson-Darling statistic	0.287	*p* > 0.05	Residuals normally distributed	✓ Pass
Shapiro–Wilk Test	W-statistic	0.9834	*p* = 0.6421 (>0.05)	Confirms residual normality	✓ Pass
Homoscedasticity	Levene’s Test *p*-value	0.732	>0.05	The constant variance assumption is valid	✓ Pass
Breusch-Pagan Test	χ^2^ *p*-value	0.6847	>0.05	Confirms homoscedasticity	✓ Pass
Autocorrelation	Durbin-Watson statistic	1.98	1.5 < DW < 2.5	No serial correlation in residuals	✓ Pass
Multicollinearity	Variance Inflation Factor (VIF)	1.12–2.34	<10 (ideally <5)	No collinearity among predictors	✓ Pass
Outlier Detection	Cook’s Distance (max)	0.142	<1.0 (ideally <0.5)	No influential outliers detected	✓ Pass
Leverage Analysis	Hat values (max)	0.387	<3(*p* + 1)/*n* = 0.404	No high-leverage points	✓ Pass
Residual Independence	Runs Test *p*-value	0.5834	>0.05	Residuals randomly distributed	✓ Pass
Model Parsimony	Akaike Information Criterion (AIC)	247.3	Lower is better	Optimal model complexity	✓ Pass
Bayesian Information Criterion	BIC	289.6	Lower is better	Balances fit and parsimony	✓ Pass
Predicted Residual Sum of Squares	PRESS	612.4	Lower is better	Cross-validation error minimal	✓ Pass
Mean Absolute Error	MAE (mg g^−1^)	2.18	Lower is better	Average prediction error is low	✓ Pass
Mean Absolute Percentage Error	MAPE (%)	2.64	<10% (excellent)	High prediction accuracy	✓ Pass

**Table 7:** Thermodynamic factors for Be^2+^ ions adsorption by TFP-HEDA at different temperatures.

ΔH° (kJ mol^−1^)	ΔS° (J mol^−1^ K^−1^)	ΔG° (kJ mol^−1^)				
9.374	59.89	298 K	313 K	323 K	333 K	343 K
		−17.839	−18.736	−19.335	−19.374	−20.532

**Table 8:** Tolerance limits of foreign ions on Be^2+^ recovery by TFP-HEDA. *Conditions*: [Be^2+^] = 10 mg L^−1^; pH 5.0; adsorbent dose = 60 mg/100 mL; T = 25 °C; contact time = 30 min; n = 3. Tolerance limit = maximum C_foreign_/C_Be_^2+^ (*w*/*w*) yielding ≤5% relative error in Be^2+^ recovery.

Foreign Ion	Charge	Ionic Radius (Å)	Tolerance Limit (*w*/*w*)	Max Error (%)
NO_3_^−^, Cl^−^	−1	—/1.81	10,000	≤0.4
Na^+^, K^+^	+1	1.02/1.38	5000	≤2.1
SO_4_^2−^	−2	—	5000	≤1.2
Li^+^, NH_4_^+^	+1	0.76/1.48	3000	≤2.4
Ba^2+^, Sr^2+^, Ca^2+^	+2	1.42–1.00	2000	≤3.1
Mg^2+^	+2	0.72	1500	≤2.8
Mn^2+^	+2	0.83	500	≤3.2
PO_4_^3−^	−3	—	500	≤3.5
Zn^2+^, Co^2+^	+2	0.74/0.75	300	≤3.8
Ni^2+^, Fe^2+^	+2	0.69/0.78	200	≤4.1
F^−^	−1	1.33	200	≤4.2
Cd^2+^	+2	0.95	150	≤4.3
Cu^2+^, Pb^2+^	+2	0.73/1.19	100	≤4.7
Al^3+^	+3	0.535	50	≤4.8
Fe^3+^	+3	0.645	30	≤4.9
Cr^3+^	+3	0.615	20	≤4.8

**Table 9:** Comparison of beryllium sorption properties for selected sorbents.

Adsorbent	Adsorption Capacity	Reference
Calcium sulfate hydrate-encapsulated silica	147.55	[90]
Aerobic granule	14	[91]
MXene/nZVI@FH	95.2	[92]
Carboxymethyl chitosan sodium form (Na- CMC)	44.96	[93]
Modified polystyrene	16	[94]
Modified Chitosan/silica	5	[95]
modified sulfonated chitosan	40	[96]
TFP-HEDA	163.67	This study

**Table 10:** Elemental composition of beryllium-containing sludge samples.

	Na_2_O	SiO_2_	Fe_2_O_3_	P_2_O_5_	CaO	MgO	MnO	LOI
wt(%)	1.25	23.78	0.782	0.23	30.51	0.863	0.018	23.92
	Contents of the main elements in BCS measured by ICP							
Element	Be		Al		Mg		Ca	
wt(%)	0.183		0.55		0.15		21.02	

## Data Availability

The original contributions presented in this study are included in the article. Further inquiries can be directed to the corresponding author.
